# Interventions to Improve Life Participation in Kidney Transplant Recipients: A Systematic Review

**DOI:** 10.1016/j.xkme.2025.100980

**Published:** 2025-02-13

**Authors:** Patrizia Natale, Angela Ju, Martin Howell, Germaine Wong, Armando Teixeira-Pinto, Anastasia Hughes, Chandana Guha, Amanda Sluiter, Nicole Scholes-Robertson, Jonathan C. Craig, Michelle A. Josephson, Giovanni Strippoli, Allison Jaure

**Affiliations:** 1 Sydney School of Public Health, The University of Sydney, Sydney, Australia; 2 Department of Precision and Regenerative Medicine and Ionian Area (DIMEPRE-J), University of Bari Aldo Moro, Bari, Italy; 3 Centre for Kidney Research, The Children’s Hospital at Westmead, Westmead, Australia; 4 Menzies Centre for Health Policy and Economics, The University of Sydney, Sydney Australia; 5 Renal Medicine, Westmead Hospital, Westmead, Australia; 6 College of Medicine and Public Health, Flinders University, Adelaide, Australia; 7 Department of Medicine, University of Chicago, Chicago, IL

**Keywords:** Kidney transplantation, life participation, quality of life, randomized controlled study, systematic review

## Abstract

**Rationale & Objective:**

Life participation, defined as the ability to participate in meaningful activities of daily living, is a critically important outcome for kidney transplant recipients. We aimed to evaluate the effectiveness of any interventions on life participation in kidney transplant recipients.

**Study Design:**

A systematic review of randomized controlled studies.

**Study Populations:**

Adult kidney transplant recipients.

**Search Strategy & Sources:**

MEDLINE, Embase, CENTRAL, PsycINFO and CINAHL were searched up to March 2023.

**Data Extraction:**

Two authors independently screened titles and abstracts, and extracted data from the included studies using standard data extraction forms.

**Analytical Approach:**

We used random-effects models with relative risk for dichotomous outcomes and mean difference for continuous outcomes with 95% confidence intervals (CIs). Confidence in the evidence was assessed using the Grading of Recommendations, Assessment, Development and Evaluation (GRADE) approach.

**Results:**

From 14,162 reports, only 33 studies (4,857 participants) were included. The risk of bias was adjudicated as high or unclear for most domains. No studies reported the outcome of life participation specifically. Among 33 studies, mental, physical and social functioning were reported in 5 (15%), 5 (15%), and 11 (33%) studies, respectively.

**Limitations:**

A wide range of interventions were included across the studies with a limited follow-up, and we were unable to pool the data and perform meta-analysis for outcomes that were reported in a single study only or in studies reporting no events.

**Conclusions:**

The effects of prebiotics, erythropoietin-stimulating agents, immunosuppressive treatments, induction therapy of interleukin-2 receptor antagonist, exercise, nutrition, education, and surgical procedures on life participation-related outcomes were uncertain. Life participation was not reported as a specific outcome in trials in kidney transplant recipients with very limited evidence on interventions for life participation-related outcomes. Trial-based evidence for interventions to improve life participation, a critical outcome for kidney transplant recipients, is needed.

## Key Learning Points

**What was known:** Life participation is considered a core outcome for kidney transplant recipients. However, the efficacy of pharmacological and nonpharmacological interventions on life participation remain uncertain in this setting.

**This study adds:** In total, 33 studies were included in this systematic review of randomized controlled studies, but none of them reported life participation. The risk of bias was assessed as high or unclear for all domains. The effects of any intervention on life participation-related outcomes were uncertain, using a wide range of interventions that prevented our capacity to perform a meta-analysis.

**Potential impact:** The evidence to inform decision-making on improving life participation in kidney transplant recipients is poor. Involving patients to address life participation in future trials is needed, focusing research on patient-centered care and improve health outcomes.

Immunosuppression is critical to maintaining graft longevity and prevent acute rejection, but the adverse effects and complications associated with immunosuppression such as tremor, diarrhea, and opportunistic infections can severely impair the overall quality of life (QoL) and life participation in kidney transplant recipients.^1^, ^2^, ^3^, ^4^, ^5^, ^6^ Apart from long-term immunosuppression use, other factors such as kidney failure, graft dysfunction, cardiac or other extra-renal complications, can limit life participation in transplant recipients.^7^

Life participation is defined as the ability to participate in meaningful activities of daily living (eg, working, studying, socializing) and has been identified by patients, caregivers, and health professionals to be a critically important outcome to be reported in all trials in kidney transplant recipients.^8^, ^9^, ^10^ However, life participation is infrequently and inconsistently reported in research with kidney transplant recipients.^11^ A recent systematic review of 230 trials and observational studies in kidney transplant recipients showed that 29 different measures were used to assess life participation, and more than half (59%) were within broader measures (eg, QoL) that included a subscale or item that captured concepts related to life participation.^11^ Typically, life participation has been captured or inferred as part of a wide range of similar outcomes, including “activities of daily living,”^12^ “social function,”^13^ and “social participation.”^14^

The current evidence on the effectiveness of interventions for improving life participation in kidney transplant recipients is limited and highly uncertain.^11^^,^^15^, ^16^, ^17^, ^18^ The aim of this systematic review was to evaluate the effectiveness of any interventions (pharmacological and nonpharmacological) on life participation in kidney transplant recipients, to inform strategies, and to improve this critical outcome in this population.

## Methods

### Study Design

We registered the protocol of this systematic review in the International Prospective Register of Systematic Reviews (PROSPERO; CRD42023412124). The study was reported according to the Preferred Reporting Items for Systematic Reviews and Meta-analysis (PRISMA) guidelines.^19^

### Search Strategy and Selection Criteria

MEDLINE, Embase, the Cochrane Central Register of Controlled Trials (CENTRAL), PsycINFO, and CINAHL were searched from inception to March 21, 2023, without language restriction (Table S1). Reference lists of review articles were also searched.

The inclusion criteria were randomized controlled trials (RCTs), quasi-RCTs, cluster RCTs or crossover studies if they evaluated the effects of any intervention (pharmacological and nonpharmacological) on life participation compared with placebo, no treatment, standard care, or any other type of intervention with adult kidney transplant recipients. Life participation was defined as the ability to participate in key activities of daily living including work, study, family, travel, hobbies, recreational and social activities or return to “normal” life and having a sense of purpose.^20^ We recognized that life participation is often included as a subscale/item within broader measures (ie, QoL). For example the EuroQol 5 Dimension (EQ-5D) assesses QoL includes “usual activities” as a subscale/item in the measure^11^ and was closely related to other broader measures (eg, mental, physical, and social functioning). Therefore, to be comprehensive, we included studies that included broader measures that explicitly included at least one subscale or item that included an aspect of life participation, and measures that captured a concept related to life participation: mental functioning, physical functioning, and social functioning.^21^, ^22^, ^23^ Condition specific measures (eg, Gastrointestinal QoL Index (GIQLI)) that included items relating to how the symptom/condition effects life participation or life participation-related outcomes (eg, usual activities) were also included. We included abstract citations only if data on life participation or any life participation-related outcomes were reported, and studies reporting a mixed population if more than 50% of the population comprised kidney transplant recipients.

Studies were excluded based on the following: participants with chronic kidney disease (CKD) without a kidney transplant (ie, CKD stages 1-5 not receiving kidney replacement therapy, CKD stage 5D receiving dialysis) and children aged under 18 years because life participation can be measured differently depending on age, sex, and severity of kidney failure, and these populations would be addressed in a separate review.

### Data Extraction

Two authors (PN, AH) independently screened the titles and abstracts of retrieved citations to identify eligible studies. Differences were resolved by consensus. Any potentially relevant citation was retrieved in full-text. Data extraction was carried out independently by 2 authors (PN, AH) using standard data extraction forms, including study design, country, sample size, participant characteristics (eg, age, sex including male and female, comorbid conditions), follow-up duration, interventions, and outcomes. Differences were resolved with discussion with a third author (AJ). Non-English studies were also eligible. When possible, corresponding authors were contacted to obtain further data. For studies published more than once, we included data from all sources, and extracted the most complete data. For crossover studies, we extracted data at the end of the first period of treatment. For cluster RCTs, we extracted data that account for the clustering in the results. Our outcomes were life participation and life participation-related outcomes.

### Risks of Bias and Quality Assessment

Two authors assessed the risk of bias in the included studies using the Cochrane Risk of Bias tool.^24^ The Grading of Recommendations, Assessment, Development and Evaluation (GRADE) approach was used to appraise the certainty of evidence, assessed as high, moderate, low, or very low certainty evidence.^25^

### Statistical Analysis

Treatment effects were estimated by random-effects pairwise meta-analysis using RevMan 5. We used relative risk (RR) for dichotomous outcomes, mean difference (MD) for continuous outcomes reported on the same scale, or standardized mean difference when continuous outcomes were reported on different scales with corresponding 95% confidence intervals (CIs). Studies analyzing change scores were reported separately in the meta-analysis from those reporting endpoint outcome data. Missing standard deviations were calculated from standard errors. Heterogeneity was first assessed with visual inspection of the forest plot. We examined the magnitude of heterogeneity variance using tau squared (τ^2^) as an indicator of the extent of heterogeneity among included studies in terms of the range of expected treatment estimates.^26^ The I^2^ statistic was also used to quantify the percentage of variation across the studies because of heterogeneity. We used the following guide for interpretation of I^2^ values: 0% to 39%, minimal heterogeneity; 40 to 79%, moderate heterogeneity; and 80% to 100%, substantial heterogeneity. We evaluated asymmetries in the funnel plots if at least 10 studies were reported for the selected outcome.

### Subgroup and Sensitivity Analyses

Subgroup analysis was performed to explore potential sources of heterogeneity by population characteristics (sex, age, comorbid conditions, type of donor), study characteristics (study design, follow-up duration), and intervention characteristics (type, doses, and route of intervention). Sensitivity analyses were performed to explore the robustness of findings, repeating the analysis taking account of risk of bias, and repeating the analysis excluding any very long or large studies to establish how much they dominate the results.

## Results

### Included Studies

**Figure 1:**
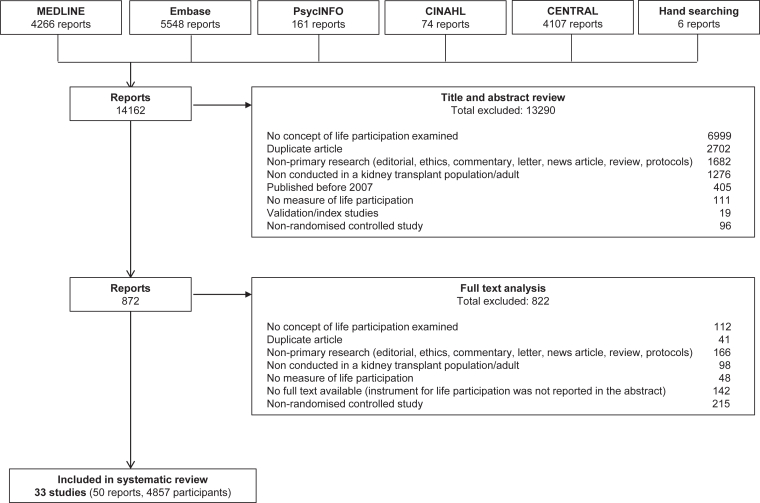
Search results.

The search strategy identified 14,162 reports. Overall, 33 RCTs (50 reports, 4,857 participants) were included (Fig 1). All studies were parallel RCTs, conducted in 13 countries (Australia (2 studies), Brazil (1 study), Canada (1 study), China (2 studies), Czech Republic (1 study), Iran (1 study), the Netherlands (1 study), New Zealand (1 study), Norway (2 studies), Spain (3 studies), Taiwan (1 study), the United Kingdom (4 studies), and the United States (8 studies)), 3 studies were multinational, and 2 studies did not report the country. The studies were conducted between 1997 and 2022, with a mean duration of 12 months. The mean age of the participants was 49.0 ± 4.2 years, and 60% were men. The characteristics of the included studies are provided in Table 1 and Table S2.

**Table 1:** Summary of Characteristics of Included Studies

Characteristic	
Number of participants	4,857
Male, n (%)	2,914 (60)
Age, y (mean, SD)	49.0 (4.2)
Type of intervention, n (%)	
Pharmacological	17 (51.5)
Nonpharmacological	16 (48.5)
Type of donor, n (%)	
Deceased	2 (6.1)
Living	2 (6.1)
Both	11 (33.4)
Country^a^, n (%)	
Australia	2 (6.1)
China	2 (6.1)
Norway	2 (6.1)
Spain	3 (9.1)
UK	4 (12.1)
US	8 (24.2)
Multinational	3 (9.1)
Other^a^	7 (21.2)
PROM^b^, n (%)	
SF-36	23 (69.7)
EQ-5D	4 (12.1)
SF-12	1 (3.0)
KDQOL-SF	1 (3.0)
MEMPHIS survey	1 (3.0)
PROMIS-29	1 (3.0)
GHQ-28	1 (3.0)
PROMIS Global Health SF	1 (3.0)
QoL-RT	1 (3.0)
GIQLI	1 (3.0)
PSWBI	2 (6.1)
Unspecified measure for QoL scale assessing LP	1 (3.0)

*Note:* percentage could be lower than 100% because of missing data in the reporting studies.

Abbreviations: QoL, quality of life; LP, life participation; PROM, Patient-Reported Outcome Measure; M, male; NS, Not stated; UK, United Kingdom; US, United States; SF-12, 12-Item Short Form Health Survey; SF-36, 36-Item Short Form Health Survey; EQ-5D, EuroQol-5D; GIQLI, Gastrointestinal Quality of Life Index (GIQLI); PGWBI, Psychological General Wellbeing Index; KDQOL-SF, Kidney Disease Quality of Life-Short Form; GHQ-28, General Health Questionnaire-28; PROMIS Global Health SF, Patient-Reported Outcomes Measurement Information System Global Health Short Form; PROMIS-29, Patient-Reported Outcomes Measurement Information System 29 Items; QoL-RT, Quality of Life Renal Transplant Recipient.

a One study each for Brazil, Canada, Czech Republic, Iran, the Netherlands, New Zealand, and Taiwan.

b Some studies reported more than one PROM.

### Interventions

The types of interventions varied across studies. Seventeen (51.5%) studies evaluated pharmacological interventions, and 16 (48.5%) studies evaluated nonpharmacological interventions. The pharmacological interventions assessed included angiotensin-converting enzyme inhibitors (ACEi), calcium-channel blockers, erythropoietin-stimulating agents (ESA), immunosuppressive therapy (including cyclosporin A (CsA), mycophenolate mofetil (MMF), tacrolimus (Tac), sirolimus (SRL), and azathioprine), thymoglobulin or induction therapy of interleukin-2 receptor antagonist (IL-2 RA). The nonpharmacological interventions assessed included prebiotics, exercise, nutrition, education (ie, behavioral contact intervention, nursing intervention based on belief model [providing manual and detailed training or care model]), pharmacy-based cyclosporin therapeutic drug monitoring service, expressive emotional education, or recording education. The surgical interventions assessed included stent or no-stent ureteroneocystostomy. The full list of interventions are listed in Table S3. No study evaluated life participation as a distinct and defined outcome.

### Risks of Bias

**Figure 2:**
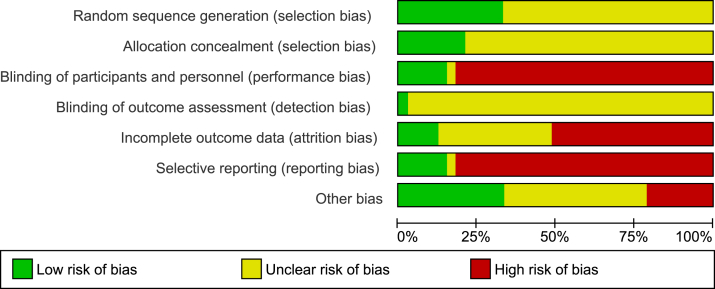
Risk of bias graph.

The risk of bias was adjudicated as high or unclear for the majority of domains, particularly in selective reporting, as shown in Figure 2 and Table S4. Overall, 11 (33.3%) studies were at low risk of bias for random sequence generation, 7 (21.2%) studies were at low risk of bias for allocation concealment, 6 (18.2%) studies reported blinding of participants and investigators, 1 (3.0%) study only reported blinding of outcome assessment, 4 (12.1%) studies were judged at low risk of bias for incomplete outcome data, 4 (12.1%) studies were at low risk of bias for selective outcome reporting, and 12 (36.4%) studies were at low risk of bias for other sources of bias (eg, funding was not involved in data analysis and interpretation).

### Patient-reported Outcome Measures

Among 33 included studies, no tools were specifically designed to assess life participation and were validated in the kidney transplant recipients, and the measures used that include life participation or life participation-related outcomes varied. Five (15.2%) studies used more than one measure. Fourteen (42.4%) studies included a broad multidomain measure that clearly reported data on specific items related to life participation-related outcomes. Among 33 studies, mental, physical, and social functioning were reported in 5 (15%), 5 (15%), and 1 (33%) studies, respectively. The broad measures that had least one subscale for life participation included the SF-36 (23 studies), the EQ-5D (4 studies), the 12-Item Short Form Health Survey (SF-12, one study), the Kidney Disease Quality of Life-Short Form (KDQOL-SF, 1 study), the MEMPHIS survey (1 study), the Patient-Reported Outcomes Measurement Information System 29 Items (PROMIS-29, 1 study), the General Health Questionnaire-28 (GHQ-28, 1 study), the Patient-Reported Outcomes Measurement Information System Global Health Short Form (PROMIS Global Health SF, 1 study), the QoL Renal Transplant Recipients (QoL-RT, 1 study), Gastrointestinal QoL Index (GIQLI, 1 study), Psychological General Wellbeing Index (PGWBI, 2 studies), or an unspecified measure for QoL scale assessing life participation (LP) (1 study). No studies used a measure designed specifically to assess ability for kidney transplant recipients to participate in life (or usual) activities. The main results for LP and life participation-related outcomes are presented in Tables 2 and 3.

**Table 2:** Summary of Findings Table for Pharmacological Interventions for Life Participation

Intervention	Outcome	Illustrative Comparative Risks (95% CI)		Relative Effect (95% CI)	Number Of Participants (Studies)	GRADE
		Assumed Risk (Control Group)	Corresponding Risk (Intervention Group)			
ESA versus no treatment	Change in quality of life (SF-36)	The mean change in quality of life score was 6.6 in the no treatment group	The mean change in quality of life score was 2.8 in the ESA group	MD –3.80 (95% CI –12.36 to 4.76)	55 participants (1 study)	Very low^a^^,^^b^^,^^c^
Cyclosporine versus fusion protein (belatacept)	Social functioning (SF-36)	The mean social functioning score was 47.6 in the belatacept group	The mean social functioning score was 46.6 in the cyclosporin group	MD −1.00 (95% CI –2.50 to 0.50)	629 participants (1 study)	Low certainty^a^^,^^b^
	Change in social functioning (SF-36)	The mean change in social functioning score across the belatacept groups ranged from 3.8 to 6.3	The mean change in social functioning score across the cyclosporin groups ranged from 1.4 to 5.1	MD –1.74 (95% CI –2.91 to –0.57)	1,048 participants (2 studies)	Low certainty^a^^,^^b^
	Mental functioning (SF-36)	The mean mental functioning score was 48.5 in the belatacept group	The mean mental functioning score was 46.9 in the cyclosporin group	MD –1.60 (95% CI–3.23 to 0.03)	622 participants (1 study)	Low certainty^a^^,^^b^
	Change in mental functioning (SF-36)	The mean change in mental functioning score was 4.8 in the belatacept group	The mean change in mental functioning score was 2.6 in the cyclosporin group	MD –2.20 (95% CI –2.34 to –2.06)	584 participants (1 study)	Low certainty^a^^,^^b^
	Physical functioning (SF-36)	The mean physical functioning score was 49.0 in the belatacept group	The mean physical functioning score was 47.1 in the cyclosporin group	MD –1.90 (95% CI –3.23 to –0.57)	622 participants (1 study)	Low certainty^a^^,^^b^
	Change in physical functioning (SF-36)	The mean change in physical functioning score was 6.3 in the belatacept group	The mean change in physical functioning score was 4.9 in the cyclosporin group	MD –1.41 (95% CI –1.53 to –1.29)	584 participants (1 study)	Low certainty^a^^,^^b^
Standard-dose cyclosporine versus low- or standard-dose tacrolimus	Social functioning (SF-36)	The mean social functioning score was 86.4 in the low-dose tacrolimus group	The mean social functioning score was 77.5 in the cyclosporin group	MD –8.90 (95% CI–25.62 to 7.82)	21 participants (1 study)	Very low^a^^,^^b^^,^^c^
	Role - emotional (SF-36)	The mean social functioning score was 84.8 in the low-dose tacrolimus group	The mean social functioning score was 55.6 in the cyclosporin group	MD –29.20 (95% CI–67.22 to 8.82)	20 participants (1 study)	Very low^a^^,^^b^^,^^c^
	Role–physical (SF-36)	The mean social functioning score was 87.5 in the low-dose tacrolimus group	The mean social functioning score was 56.3 in the cyclosporin group	MD –31.20 (95% CI –68.73 to 6.33)	18 participants (1 study)	Very low^a^^,^^b^^,^^c^
Cyclosporine + sirolimus + steroids versus sirolimus + steroids	Social functioning (SF-36)	The mean social functioning score was 85.6 in the sirolimus + steroids group	The mean social functioning score was 79.1 in the cyclosporin + sirolimus + steroids group	MD –6.50 (95% CI –12.29 to –0.71)	357 participants (1 study)	Very low^a^^,^^b^^,^^c^
	Role–emotional (SF-36)	The mean social functioning score was 84.4 in the sirolimus + steroids group	The mean social functioning score was 81.6 in the cyclosporin + sirolimus + steroids group	MD –2.80 (95% CI –11.90 to 6.30)	352 participants (1 study)	Very low^a^^,^^b^^,^^c^
	Role–physical (SF-36)	The mean social functioning score was 74.8 in the sirolimus + steroids group	The mean social functioning score was 66.9 in the cyclosporin + sirolimus + steroids group	MD –7.90 (95% CI –18.41 to 2.61)	353 participants (1 study)	Very low^a^^,^^b^^,^^c^
Enteric-coated mycophenolate sodium versus mycophenolate mofetil	Quality of life (PGWBI)	The mean quality of life score was 66.9 in the MMF group	The mean quality of life functioning score was 73.4 in the EC-MPS group	MD 6.50 (95% CI –1.05 to 14.05)	107 participants (1 study)	Very low^a^^,^^b^^,^^c^
	Change in physical functioning (SF-36)	The mean change in physical functioning score was 0.1 in the MMF group	The mean change in physical functioning score was 2.7 in the EC-MPS group	MD 2.60 (95% CI –2.67 to 7.87)	129 participants (1 study)	Very low^a^^,^^b^^,^^c^
Thymoglobulin versus IL-2 RA	Decrease in social functioning (SF-36)	354 per 1,000	195 fewer per 1,000 (273 fewer to 39 fewer)	RR 0.45 (95% CI 0.23 to 0.89)	111 participants (1 study)	Very low^a^^,^^b^^,^^c^
	Role–emotional (SF-36)	479 per 1,000	225 fewer per 1,000 (326 fewer to 53 fewer)	RR 0.53 (95% CI 0.32, 0.89)	111 participants (1 study)	Very low^a^^,^^b^^,^^c^
	Role–physical (SF-36)	479 per 1,000	29 more per 1,000 (134 fewer to 264 more)	RR 1.06 (95% CI 0.72 to 1.55)	111 participants (1 study)	Very low^a^^,^^b^^,^^c^
Steroid versus nonsteroidal treatment	Role–emotional (SF-36)	The mean role emotional score was 91.7 in the nonsteroidal treatment group	The mean role emotional score was 77.7 in the steroid group	MD –14.00 (95% CI–38.89 to 10.89)	23 participants (1 study)	Very low^a^^,^^b^^,^^c^
	Mental functioning (SF-36)	The mean mental functioning score was 56.0 in the nonsteroidal treatment group	The mean mental functioning score was 52.3 in the steroid group	MD –3.70 (95% CI –8.96 to 1.56)	23 participants (1 study)	Very low^a^^,^^b^^,^^c^
	Role–physical (SF-36)	The mean role emotional score was 75.0 in the nonsteroidal treatment group	The mean role emotional score was 63.9 in the steroid group	MD –11.10 (95% CI–45.77 to 23.57)	23 participants (1 study)	Very low^a^^,^^b^^,^^c^
	Physical functioning (SF-36)	The mean physical functioning score was 48.4 in the nonsteroidal treatment group	The mean physical functioning score was 42.6 in the steroid group	MD –5.80 (95% CI –14.31 to 2.71)	23 participants (1 study)	Very low^a^^,^^b^^,^^c^
	Social functioning (SF-36)	The mean social functioning score was 84.4 in the nonsteroidal treatment group	The mean social functioning score was 68.0 in the steroid group	MD –16.40 (95% CI –33.78 to 0.98)	23 participants (1 study)	Very low^a^^,^^b^^,^^c^

*Note:* The reduction in mean for SF-36 implied a worsen in the outcome.

Abbreviations: MD, mean difference; RR: relative risk; CI, confidence intervals; ESA, erythropoietin-stimulating agents; SF-36, 36-Item Short Form Health Survey; PGWBI, Psychological General Wellbeing Index; LP, life participation; MMF, mycophenolate mofetil; EC-MPS, enteric-coated mycophenolate sodium; IL-2 RA, interleukin-2 receptor antagonist.

a Downgraded of one level because of study limitation.

b Downgraded of one level because of imprecision.

c Downgraded of one level because a single study reported the outcome and optimal information size (OIS) not met.

**Table 3:** Summary of Findings Table for Nonpharmacological Interventions for Life Participation

Intervention	Outcome	Illustrative Comparative Risks (95% CI)		Relative Effect (95% CI)	Number of participants (studies)	GRADE
		**Assumed Risk (Control Group)**	**Corresponding Risk (Intervention Group)**			
Prebiotics versus placebo/control	Change in quality of life (EQ-5D)	The mean change in quality of life score was −0.3 in the placebo group	The mean change in quality of life score was −0.2 in the prebiotic group	MD 0.10 (95% CI –0.00 to 0.20)	56 participants (1 study)	Low certainty^a^
Exercise versus standard care	Change in social functioning (SF-36)	The mean change social functioning score was −9.8 in the standard care group	The mean change in social functioning score was 15.2 in the exercise group	MD 25.02 (95% CI 9.41 to 40.63)	31 participants (1 study)	Very low^b^^,^^c^^,^^d^
	Change in overall quality of life (SF-36)	The mean change in overall quality of life score was −3.4 in the standard care group	The mean change in overall quality of life score was 8.6 in the exercise group	MD 12.00 (95% CI 3.02 to 20.98)	31 participants (1 study)	Very low^b^^,^^c^^,^^d^
	Mental functioning (SF-36)	The mean mental functioning score was 55.7 in the standard care group	The mean mental functioning score was 52.2 in the exercise group	MD –3.50 (95% CI –11.17 to 4.17)	33 participants (1 study)	Very low^b^^,^^c^^,^^d^
	Change in mental functioning (SF-36)	The mean change in mental functioning score was −9.4 in the standard care group	The mean change in mental functioning score was −6.9 in the exercise group	MD 2.50 (95% CI –21.41 to 26.41)	31 participants (1 study)	Very low^b^^,^^c^^,^^d^
	Physical functioning (SF-36)	The mean physical functioning score was 41.3 in the standard care group	The mean physical functioning score was 37.8 in the exercise group	MD –3.50 (95% CI –8.84 to 1.84)	33 participants (1 study)	Very low^b^^,^^c^^,^^d^
Aerobic exercise versus resistance training	Mental functioning (SF-36)	The mean mental functioning score was 51.5 in the resistance training group	The mean mental functioning score was 52.2 in the aerobic exercise group	MD 0.70 (95% CI –10.23 to 11.63)	26 participants (1 study)	Very low^b^^,^^c^^,^^d^
	Physical functioning (SF-36)	The mean physical functioning score was 43.5 in the resistance training group	The mean physical functioning score was 37.8 in the aerobic exercise group	MD –5.70 (95% CI –17.20 to 5.80)	26 participants (1 study)	Very low^b^^,^^c^^,^^d^
Resistance training versus standard care	Satisfaction with social role (PROMIS-29)	The mean satisfaction with social role score was 52.8 in the standard care group	The mean satisfaction with social role score was 54.0 in the resistance training group	MD 1.21 (95% CI –2.94 to 5.36)	80 participants (1 study)	Very low^b^^,^^c^^,^^d^
	Quality of life (SF-36)	The mean quality of life score was 43.6 in the standard care group	The mean quality of life score was 58.3 in the resistance training group	MD 14.70 (95% CI –2.75 to 32.15)	17 participants (1 study)	Very low^b^^,^^c^^,^^d^
	Mental functioning (PROMIS-29)	The mean mental functioning score across the standard care groups ranged from 49.5 to 55.7	The mean mental functioning score across the resistance training groups ranged from 51.5 to 53.1	MD 0.07 (95% CI –0.78 to 0.93)	113 participants (1 study)	Very low^b^^,^^c^^,^^d^^,^^e^
	Physical functioning (PROMIS-29)	The mean physical functioning score across the standard care groups ranged from 41.3 to 46.6	The mean physical functioning score across the resistance training groups ranged from 43.5 to 51.6	MD 0.45 (95% CI 0.05 to 0.86)	113 participants (1 study)	Very low^b^^,^^c^^,^^d^
Intense nutrition versus standard nutrition	Social functioning (SF-36)	The mean social functioning score was 92.3 in the standard nutrition group	The mean social functioning score was 91.7 in the intense nutrition group	MD –0.60 (95% CI –11.38 to 10.18)	36 participants (1 study)	Very low^b^^,^^c^^,^^d^
	Role–emotional (SF-36)	The mean social functioning score was 94.9 in the standard nutrition group	The mean social functioning score was 93.1 in the intense nutrition group	MD –1.80 (95% CI –12.04 to 8.44)	36 participants (1 study)	Very low^b^^,^^c^^,^^d^
	Role–physical (SF-36)	The mean social functioning score was 92.8 in the standard nutrition group	The mean social functioning score was 78.8 in the intense nutrition group	MD –14.0 (95% CI –29.14 to 1.14)	36 participants (1 study)	Very low^b^^,^^c^^,^^d^
Expressive emotion education versus recording education without emotion disclosure	Social dysfunction (GHQ-28)	The mean social dysfunction score was 3.2 in the recording education group	The mean social dysfunction score was 1.2 in the expressive emotion education group	MD –1.96 (95% CI –2.60 to –1.32)	64 participants (1 study)	Very low^b^^,^^c^^,^^d^
	Quality of life (GHQ-28)	The mean quality of life score was 14.5 in the recording education group	The mean quality of life score was 8.1 in the expressive emotion group	MD –6.44 (95% CI –8.79 to –4.09)	64 participants (1 study)	Very low^b^^,^^c^^,^^d^
Education versus standard care	Social functioning (unspecified QoL scale assessing LP)	The mean social functioning score was 16.6 in the standard care group	The mean social functioning score was 12.1 in the education group	MD –4.43 (95% CI –6.10 to –2.76)	60 participants (1 study)	Very low^b^^,^^c^^,^^d^
Steroid versus nonsteroidal treatment	Role–emotional (SF-36)	The mean role emotional score was 91.7 in the nonsteroidal treatment group	The mean role emotional score was 77.7 in the steroid group	MD –14.00 (95% CI –38.89 to 10.89)	23 participants (1 study)	Very low^b^^,^^c^^,^^d^
	Mental functioning (SF-36)	The mean mental functioning score was 56.0 in the nonsteroidal treatment group	The mean mental functioning score was 52.3 in the steroid group	MD –3.70 (95% CI –8.96 to 1.56)	23 participants (1 study)	Very low^b^^,^^c^^,^^d^
	Role - physical (SF-36)	The mean role emotional score was 75.0 in the nonsteroidal treatment group	The mean role emotional score was 63.9 in the steroid group	MD –11.10 (95% CI –45.77 to 23.57)	23 participants (1 study)	Very low^b^^,^^c^^,^^d^
	Physical functioning (SF-36)	The mean physical functioning score was 48.4 in the nonsteroidal treatment group	The mean physical functioning score was 42.6 in the steroid group	MD –5.80 (95% CI –14.31 to 2.71)	23 participants (1 study)	Very low^b^^,^^c^^,^^d^
	Social functioning (SF-36)	The mean social functioning score was 84.4 in the nonsteroidal treatment group	The mean social functioning score was 68.0 in the steroid group	MD –16.40 (95% CI –33.78 to 0.98)	23 participants (1 study)	Very low^b^^,^^c^^,^^d^

*Note:* The reduction in mean for EQ-5D, SF-36, PROMIS-29, Patient-Reported Outcomes Measurement Information System 29 Items and GHQ-28 implied a worsen in the outcome.

Abbreviations: MD, mean difference; RR: relative risk; CI, confidence intervals; SF-36, 36-Item Short Form Health Survey; EQ-5D, EuroQol-5D; PROMIS-29, Patient-Reported Outcomes Measurement Information System 29 Items; GHQ-28, General Health Questionnaire-28; QoL, quality of life; LP, life participation; LP, life participation.

a Downgraded of 2 levels because a single study reported the outcome and optimal information size (OIS) not met.

b Downgraded of one level because of study limitation.

c Downgraded of one level because of imprecision.

d Downgraded of one level because a single study reported the outcome and optimal information size (OIS) not met.

e Downgraded of one level because of inconsistency (moderate heterogeneity).

### Outcomes

#### QoL

In a RCT comparing prebiotics to placebo, there was no difference in QoL (Fig S1.1; 1 study, 56 participants: EQ-5D score; MD 0.10, 95% CI –0.00 to 0.20, low certainty evidence). The effects of ESA or exercise on change in QoL (Fig S2.1; S8.2, SF-36 score) compared with standard care/no treatment were uncertain. Enteric-coated mycophenolate sodium had uncertain effects on QoL (Fig S6.1, PGWBI) compared with mycophenolate mofetil. Resistance training had uncertain effects on QoL (Fig S10.2, SF-36 score) compared with standard care. Expressive emotion education had uncertain effects on QoL (Fig S12.2, GHQ-28) compared with education without emotion disclosure (Table 2).

#### Mental Functioning

A single study reported that cyclosporin may reduce mental functioning (Fig S3.3; 1 study, 622 participants: SF-36 score; MD –1.60, 95% CI –3.23 to 0.03), or change in mental functioning (Fig S3.4; 1 study, 584 participants: SF-36 score; MD –2.20, 95% CI –2.34 to –2.06) compared with belatacept (low certainty evidence). The effects of other immunosuppressive treatments or nutrition on mental functioning defined as “role-emotional” were uncertain (Figs S4.2, S5.2, S7.2, S11.2) (very low certainty evidence). It was uncertain whether exercise had any effects on mental functioning (Fig S8.3-4, SF-36 score) (very low certainty evidence) compared with standard care. Exercise (aerobic exercise or resistance training) had uncertain effects on mental functioning (Figs S9.1, S10.3; SF-36 score, PROMIS Global Health SF) (very low certainty evidence) compared with another type of exercise or standard care. Steroid had uncertain effects on mental functioning defined as role emotional (Figure S14.1, SF-36 score) or mental functioning (Figure S14.2, SF-36 score) (very low certainty evidence) compared with nonsteroidal treatment (Table 2).

#### Physical Functioning

A single study reported that cyclosporin may reduce physical functioning (Fig S3.5; 1 study, 622 participants: SF-36 score; MD –1.90, 95% CI –3.23 to –0.57), and change in physical functioning (Fig S3.6; 1 study, 584 participants: SF-36 score; MD –1.41, 95% CI –1.53 to –1.29) compared with belatacept (low certainty evidence). The effects of other immunosuppressive treatments or nutrition on physical functioning defined as “role-physical” were uncertain (Figs S4.3, S5.3, S7.3, S11.3) (very low certainty evidence). Enteric-coated mycophenolate sodium had uncertain effects on change in physical functioning (Fig S6.2, SF-36 score) compared with mycophenolate mofetil. It was uncertain whether exercise had any effects on physical component functioning (Fig S8.5, SF-36 score) (very low certainty evidence) compared with standard care. Exercise (aerobic exercise or resistance training) had uncertain effects on physical functioning (Figs S9.2, S10.4; SF-36 score, PROMIS Global Health SF) (very low certainty evidence) compared with another type of exercise or standard care. Steroid had uncertain effects on physical functioning defined as role–physical (Fig S14.3, SF-36 score) or physical functioning (Fig S14.4, SF-36 score) (very low certainty evidence) compared with nonsteroidal treatment (Table 2).

#### Social Functioning

A single study reported that cyclosporin may worsen social functioning (Figure S3.1; 1 study, 629 participants: SF-36 score; MD –1.00, 95% CI –2.50 to 0.50), and change in social functioning (Figure S3.2; 2 studies, 1,048 participants: SF-36 score; MD –1.74, 95% CI –2.91 to –0.57, I^2^ = 11%) compared with belatacept (low certainty evidence). The effects of other immunosuppressive treatments, nutrition, or nursing intervention on social functioning were uncertain (Figs S4.1, S5.1, S11.1, S13.1; SF-36 score, unspecified QoL scale assessing LP). Thymoglobulin had uncertain effects on social functioning (Fig S12.1, GHQ-28) compared with induction therapy of interleukin-2 receptor antagonist (IL-2 RA). It was uncertain whether exercise had any effects on change in social functioning (Fig S8.1, SF-36 score) (very low certainty evidence) compared with standard care. Expressive emotion education had uncertain effects on social dysfunction (Fig S12.1, GHQ-28) (very low certainty evidence) compared with recording education without emotion disclosure. Resistance training had uncertain effects on satisfaction with social role (Fig S10.1, PROMIS-29) compared with standard care. Steroid had uncertain effects on social functioning (Fig S14.5, SF-36 score) (very low certainty evidence) compared with nonsteroidal treatment (Table 2).

#### Sensitivity and Subgroup Analyses

Sensitivity and subgroup analyses were not possible because of insufficient data.

## Discussion

Overall, there is very limited evidence for interventions to improve LP in kidney transplant recipients. Only 33 RCTs involving 4,857 kidney transplant recipients were included in our review, but no trial reported LP as a separate or specific outcome defined as the ability to participate in meaningful activities of daily living.^10^ Instead, trials used measures of broader measures (ie, QoL) that included LP as a subscale/item or outcomes that captured the concept of LP under physical, mental, or social functioning. Across the trials, there were 11 different measures that captured an aspect of LP. The majority of trials (70%) used the SF-36 (QoL). The risk of bias was adjudicated as high or unclear for the majority of domains, particularly selective reporting. The effects of prebiotics, ESA, immunosuppressive treatments, induction therapy of IL-2 RA, exercise, nutrition, education, and surgical procedures on LP were also very uncertain.

Across other stages of CKD, there is some evidence to suggest that lifestyle interventions (including exercise, diet or education) may improve QoL; mental, physical, and social functioning; and work status. Previous systematic reviews have shown that exercise and education may improve QoL in patients with kidney disease not requiring kidney replacement therapy.^27^, ^28^, ^29^ Trials in patients receiving dialysis suggest that psychosocial interventions (such as cognitive behavioral therapy) and physical activity may improve QoL, including physical and mental functioning.^30^, ^31^, ^32^, ^33^ More broadly, in adults with other long-term chronic conditions including stroke and cancer, individual skills training or counseling, support groups, and education interventions have been shown to improve social participation and ability to do usual activities.^34^

The sparse evidence for the effect of interventions on LP in kidney transplantation may in part be due to the lack of patient-reported outcome measures for LP that have been validated in the kidney transplant population.^10^^,^^35^ Patient-reported outcome measures such as the PROMIS Ability to Participate in Social Roles and Activities^36^ and the Reintegration to Normal Living Index (RNLI)^37^ capture LP and have been used in rehabilitation, chronic heart failure, chronic obstructive pulmonary disease, chronic back pain, depression, or stroke,^38^, ^39^, ^40^ however, have not been validated or used in trials in kidney transplantation. Through the Standardized Outcomes in Nephrology (SONG) initiative, a new core outcome measures for LP has been developed and validated to support consistent assessment of LP in a way that is meaningful to patients, caregivers, and clinicians.^35^^,^^41^ This may also address the problem of selective reporting bias. Of note, a systematic review of trials of immunosuppressive agents in kidney transplant recipients found that all trials that reported QoL favored the intervention, which strongly suggests selective reporting bias.^42^

This review was carried out using a highly sensitive search strategy that included all interventions. Data extraction and analysis were performed by 2 independent reviewers, who used the risk of bias Cochrane tool and the GRADE approach to assess the quality of the evidence. However, our study had some limitations. A wide range of interventions were included across the studies with a limited follow-up, and we were unable to pool the data and perform meta-analysis for outcomes that were reported in a single study only or in studies reporting no events. Generally, the risk of bias was assessed as high or unclear in all domains limiting our certainty in the evidence. The studies included a limited number of participants and events that prevented our ability to provide firm conclusions, and prespecified subgroup and sensitivity analyses could not be performed to explore possible sources of heterogeneity. There were insufficient data to assess the safety of the interventions used to improve LP in kidney transplant recipients. Although we have included all broader measures that assessed at least one item of LP or life participation-related outcomes, it was not easy to evaluate the contribution of the LP in measures assessing QoL. Mental and physical functioning were based on different definitions, including emotional or physical roles, that prevented our ability to generalize our findings. There were insufficient data to explore selective reporting using funnel plot.

Systematic reviews conducted in other patient populations have shown that symptom management, lifestyle modification (eg, exercise), nonsurgical or cognitive interventions, and education and counseling program may improve LP.^43^, ^44^, ^45^ Symptom management strategies may be relevant as kidney transplant recipients report various symptoms including pain, muscle weakness, fatigue, graft scaring, weight gain, and cognitive impairment, which could limit LP.^46^, ^47^, ^48^, ^49^, ^50^ There is also a high prevalence of depression and anxiety in kidney transplant recipients related to potential side effects of immunosuppression; therefore, psychosocial support for patients and their families to manage distress, anxiety and depression, or tremors also have potential to improve LP in kidney transplant recipients.^51^ There is a need to develop and evaluate interventions in high quality trials, and we suggest that kidney transplant recipients and caregivers be involved in the design of interventions to maximize acceptability, uptake, and impact.^52^, ^53^, ^54^

Overall, there is very limited evidence for interventions to improve LP in kidney transplant recipients. There is wide heterogeneity in the interventions and LP and life participation-related outcomes across trials. Consequently, there is little evidence to inform shared decision-making about how to maximize LP in kidney transplant recipients. Coproduction of intervention and trials with patients to address LP are needed, which can help to ensure patient-centered care and improve health outcomes and ultimately the overall success of kidney transplantation.
