# Supplementary material for: Interventions to Improve Life Participation in Kidney Transplant Recipients: A Systematic Review
Source: Kidney Med. 2025 Feb 13;7(4):100980. doi: 10.1016/j.xkme.2025.100980 (PMC11982030; doi:10.1016/j.xkme.2025.100980)
Supplement: Supplementary File (PDF) — Figures S1-S14; Tables S1-S4. [file mmc1.pdf]

Table S1. Search Strategy

MEDLINE 1946 to March 21, 2023

1. "Quality of Life"/
2. (quality adj1 life).tw.
3. qol.tw.
4. hrqol.tw.
5. (quality and life).tw.
6. exp "severity of illness index"/ or exp sickness impact profile/
7. 1 or 2 or 3 or 4 or 5 or 6
8. exp "activities of daily living"/ or exp leisure activities/ or exp travel/ or exp work/
9. (participation or engagement or inclusion).tw.
10. (communit* or recreation* or leisure*).tw.
11. (school* or education* or class* or teach*).tw.
12. (home or house or family or families).tw.
13. (social adj participation).tw.
14. 8 or 9 or 10 or 11 or 12 or 13
15. exp employment/
16. employ.tw.
17. (return adj2 work).tw.
18. (employ* adj status).tw.
19. 15 or 16 or 17 or 18
20. 7 or 14 or 19
21. exp Kidney Transplantation/
22. 20 and 21
23. randomized controlled trial.pt.
24. controlled clinical trial.pt.
25. randomized.ab.
26. placebo.ab.

27. Clinical Trials as Topic/
28. randomly.ab.
29. (crossover or cross-over).tw.
30. trial.ti.
31. 23 or 24 or 25 or 26 or 27 or 28 or 29 or 30
32. Animals/ not (animals/ and Humans/)
33. 31 not 32
34. 22 and 33

Embase 1974 to March 21, 2023

1. "Quality of Life"/
2. (quality adj1 life).tw.
3. (quality and life).tw.
4. hrqol.tw.
5. exp "severity of illness index"/ or exp sickness impact profile/
6. qol.tw.
7. or/1-6
8. exp "activities of daily living"/ or exp leisure activities/ or exp travel/ or exp work/
9. (social adj participation).tw.

10. (participation or engagement or inclusion).tw.
11. (communit* or recreation* or leisure*).tw.
12. (home or house or family or families).tw.
13. (school* or education* or class* or teach*).tw.
14. or/8-13
15. exp employment/
16. employ.tw.
17. (return adj2 work).tw.
18. (employ* adj status).tw.
19. or/15-18
20. or/7,14,19

21. exp Kidney Transplantation/
22. or/20,21
23. randomized controlled trial.pt.
24. controlled clinical trial.pt.
25. randomized.ab.
26. placebo.ab.
27. Clinical Trials as Topic/
28. randomly.ab.
29. (crossover or cross-over).tw.
30. trial.ti.
31. or/23-30
32. Animals/ not (animals/ and Humans/)
33. 31 not 32
34. 22 and 33

CENTRAL to March 21, 2023

1. exp Kidney Transplantation/
2. (communit* or recreation* or leisure*).tw.
3. (home or house or family or families).tw.
4. (school* or education* or class* or teach*).tw.
5. (participation or engagement or inclusion).tw.
6. (social adj participation).tw.
7. exp "activities of daily living"/ or exp leisure activities/ or exp travel/ or exp work/
8. exp employment/
9. employ.tw
10. (employ* adj status).tw.
11. (return adj2 work).tw.
12. (quality adj1 life).tw.
13. qol.tw.
14. hrqol.tw.
15. (quality and life).tw.
16. "Quality of Life"/

17. exp "severity of illness index"/ or exp sickness impact profile/

18. or/2-17

19. 1 and 18

20. randomized controlled trial.pt.

21. controlled clinical trial.pt.

22. randomized.ab.

23. placebo.ab.

24. Clinical Trials as Topic/

25. randomly.ab.

26. (crossover or cross-over).tw.

27. trial.ti.

28. or/20-27

29. Animals/ not (animals/ and Humans/)

30. 28 not 29

31. 19 and 30

PsycINFO 1806 to March 21, 2023

1. "Quality of Life"/

2. (quality adj1 life).tw.

3. qol.tw.

4. hrqol.tw.

5. exp Activities of Daily Living/

6. exp Recreation/

7. (participation or engagement or inclusion).tw.

8. exp Quality of Work Life/

9. employment/

10. (social adj participation).tw.

11. (communit* or recreation* or leisure*).tw.

12. (school* or education* or class* or teach*).tw.

13. (home or house or family or families).tw.

14. (employ* adj status).tw.

15. exp Kidney Disease/

16. exp Organ Transplantation/ or exp Kidneys/

17. exp Clinical Trials/ or exp Randomized Controlled Trials/

18. randomized.ab.

19. placebo.ab.

20. (crossover or cross-over).tw.

21. trial.ti.

22. or/1-14

23. or/15,16

24. or/17-21

25. 22 and 23 and 24

CINHAL to March 21, 2023

S1. (MH "Quality of Life+") OR (MH "Health and Life Quality (Iowa NOC)+")

S2. (MH "Leisure Activities+") OR (MH "Activities of Daily Living+")

S3. (MM "Transplant Recipients") OR (MH "Kidney Transplantation+")

S4. (MH "Randomized Controlled Trials+") OR (MH "Clinical Trials+")

S5. S1 AND S2 AND S3 AND S4

Table S2. Characteristics of included studies

Study ID	Trial name/registration number	Study design	Country	Sample size	Type of intervention	Age	M (%)	Deceased donor (%)	PROM	Item related to life participation	Study duration (months)
Pharmacological intervention											
Aasebo 2005 (1)	NS	Parallel RCT	Norway	57	Lisinopril 10-20 mg	44.0±13.0	17.0	42.0	SF-36	Quality of life; social functioning (including social activity and emotional problems interfered with normal social activities); mental composite (including emotional health problems related to work or other activities, role - emotion); physical composite (including limitation in activities, role - physical)	12
				67	Nifedipine 30-60 mg	45.0±14.0	14.0	52.0			
Al-Otaibi 2019* (2)	NS	Parallel RCT	NS	183	ESA (target Hb 11-12 g/dl)	NS	31.1	NS	SF-36	Quality of life; social functioning (including social activity and emotional problems interfered with normal social activities); mental composite (including emotional health problems related to work or other activities, role - emotion); physical composite (including limitation in activities, role - physical)	12
				64	ESA (target Hb 13-15 g/dl)	NS	50.0	NS			
Anderson 2011* (3)	NS	Parallel RCT	US	207^	Alemtuzumab	49.2±3.4^	58.7^	NS	SF-36	Quality of life; social functioning (including social activity and emotional problems interfered with normal	24
					r-ATG			NS			

										social activities); mental composite (including emotional health problems related to work or other activities, role - emotion); physical composite (including limitation in activities, role - physical)	
Baltar 2002 (4)	NS	Parallel RCT	Spain	26^	AZA	41.0, SD not reported	69.0	100	PGWBI, EQ-5D	**PGWBI:** Quality of life; physical composite (related to feel healthy enough to carry out the things) satisfaction on personal life; interest in things of daily life<br>**EQ-5D:** Quality of life (including usual activities)	12
					MMF			100			
Durrbach 2010 (5)[!]	BENEFIT-EXT, NCT00114777	Parallel RCT	Multinational	184	CsA 150-300 ng/mL, orally	55.7±12.2	63.0	100	SF-36	Quality of life; social functioning (including social activity and emotional problems interfered with normal social activities); mental composite (including emotional health problems related to work or other activities, role - emotion); physical composite (including limitation in activities, role - physical)	36
				175	Belatacept (less intense) IV 10mg/Kg	56.1±12.4	73.7	100			
				184	Belatacept (more intensive) IV 10mg/Kg	56.7±12.6	64.7	100			
Kumana 2003 (6)	NS	Parallel RCT	UK	57	Diltiazem 30 or 60 mg	42.2±10.6	70.9	NS	SF-36	Quality of life; social functioning (including social activity and emotional problems interfered with normal social activities); mental composite (including emotional health problems related to	6
				57	Placebo	41.8±9.6	70.9	NS			

										work or other activities, role - emotion); physical composite (including limitation in activities, role - physical)	
Oppenheimer 2009 (7)	NS	Parallel RCT	Spain	40	Standard-dose CsA, MMF, and corticosteroids	50.4±11.0	55.0	NS	SF-36	Quality of life; social functioning (including social activity and emotional problems interfered with normal social activities); mental composite (including emotional health problems related to work or other activities, role - emotion); physical composite (including limitation in activities, role - physical)	12
				38	Daclizumab induction, MMF, and corticosteroids with a low-dose of CsA 50-100 ng/mL	50.7±11.7	68.4	NS			
				40	Low dose Tac 3-7 ng/mL	49.4±14.6	70.0	NS			
				38	Low dose sirolimus 4-8 ng/mL	46.3±12.1	47.4	NS			
Ortega 2011 (8)	MYVIDA	Parallel RCT	Spain	54	MMF	49.9±11.9	59.3	96.3	PGWBI	Quality of life; physical composite (related to feel healthy enough to carry out the things) satisfaction on personal life; interest in things of daily life	3
				59	EC-MPD	51.8±2.4	59.3	100			
Painter 2003 (9)	NS	Parallel RCT	US	15	Prednisolone + induction therapy of IL-2	46.8±4.4	78	NS	SF-36	Quality of life; social functioning (including social activity and emotional problems interfered with normal social activities); mental composite (including emotional health problems related to work or other activities, role - emotion); physical composite (including	12
				10	Non-steroidal treatment + induction therapy of IL-2	48.3±12.7	66	NS			

										limitation in activities, role - physical)	
Palanisamy 2015 (10)[!]	NCT00859131	Parallel RCT	US	102	Thymoglobulin (r-ATG 1.5 mg/kg IV)	52.0±13.0	57.8	NS	SF-36	Quality of life; social functioning (including social activity and emotional problems interfered with normal social activities); mental composite (including emotional health problems related to work or other activities, role - emotion); physical composite (including limitation in activities, role - physical)	12
				98	Induction therapy of IL-2 RA (daclizumab)	49.0±14.0	63.3	NS			
Pile 2020 (11)	ISRCTN41687085	Parallel RCT	UK	28	Epoetin beta (target Hb 11.5-13.5 g/dL)	49.0±2.0	38.0	NS	SF-36	Quality of life; social functioning (including social activity and emotional problems interfered with normal social activities); mental composite (including emotional health problems related to work or other activities, role - emotion); physical composite (including limitation in activities, role - physical)	24
				27	No treatment	47.0±2.0	33.0	NS			
Rohan 2019* (12)	BEST	Parallel RCT	NS	315^	Alemtuzumab + belatacept	NS	NS	NS	MEMPHIS Survey	Quality of life; life/role responsibilities (including participating in social activities; performing job; participating in leisure pastimes; ability to travel on vacations)	24
					r-ATG + belatacept	NS	NS	NS			
					r-ATG + Tac						
Russ 2007 (13)	NS	Parallel RCT	Multinational	215	SRL 15/30 ng/mL + corticosteroids	43.9±13.0	62.0	87.0	SF-36	Quality of life; social functioning (including social activity and	36

				215	SRL 15/30 ng/mL + CsA 50–200 ng/mL + corticosteroids	45.2±11.4	68.0	88.0		emotional problems interfered with normal social activities); mental composite (including emotional health problems related to work or other activities, role - emotion); physical composite (including limitation in activities, role - physical); apparence	
Shehata 2009 (14)	NS	Parallel RCT	UK	69	EC-MPS 440 mg/day	46.1±11.0	54.4	57.4	SF-36, GIQLI	**SF-36:** Quality of life; social functioning (including social activity and emotional problems interfered with normal social activities); mental composite (including emotional health problems related to work or other activities, role - emotion); physical composite (including limitation in activities, role - physical)	2.8
				65	MMF 2000 mg/day	49.0±13.3	59.0	75.4		**GIQLI:** daily activities (school, work, household); leisure or recreational activities; change in apparence	
Shield 1997 (15)	NS	Parallel RCT	US	412^	Tac	NS	NS	NS	SF-36	Quality of life; social functioning (including social activity and emotional problems interfered with normal social activities); mental composite (including emotional health problems related to work or other activities, role - emotion); physical	12
					CsA	NS	NS	NS			

										composite (including limitation in activities, role - physical)	
Vincenti 2010 (16)[!]	BENEFIT, NCT00256750	Parallel RCT	Multinational	221	CsA 150-300 ng/mL, orally	43.5±4.3	74.7	NS	SF-36	Quality of life; social functioning (including social activity and emotional problems interfered with normal social activities); mental composite (including emotional health problems related to work or other activities, role - emotion); physical composite (including limitation in activities, role – physical)	36
				226	Belatacept (less intense) IV 10mg/kg	42.6±13.4	64.6	NS			
				219	Belatacept (more intensive) IV 10mg/Kg	43.6±14.6	68.9	NS			
Walker 2007 (17)	NS	Parallel RCT	Australia	10	CsA 100–400 ng/mL	38.8±14.4	50.0	NS	SF-36	Quality of life; social functioning (including social activity and emotional problems interfered with normal social activities); mental composite (including emotional health problems related to work or other activities, role - emotion); physical composite (including limitation in activities, role - physical)	12
				11	Tac 5–15 ng/mL	38.6±10.4	54.5	NS			
Non-pharmacological intervention											
Aasebo 2019 (18)	ORENTRA	Parallel RCT, NCT01744067	Norway	66	Marine n-3 PUFA 2.6 g for 44 weeks	52.8 ±13.5	71.2	NS	SF-36	Quality of life; social functioning (including social activity and emotional problems interfered with normal social activities); mental composite (including emotional health problems related to	12
				66	Olive oil for 44 weeks	54.1 ±14.2	77.3	NS			

										work or other activities, role - emotion); physical composite (including limitation in activities, role - physical)	
Chan 2022 (19)	PREBIOTIC, ACTRN12618001057279	Parallel RCT	Australia	27	Prebiotics supplementation (banana–resistant starch 7.5-15 g/day) for 6 weeks	52.9±12.1	63.0	NS	EQ-5D	Quality of life (including usual activities)	6
				29	Placebo for 6 weeks	54.7±10.9	62.0	NS			
Chisholm-Burns 2011* (20)		Parallel RCT	US	71^	Behavioral contract intervention	50.0±12.4^	NS	60.6^	SF-12, EQ-5D	**SF-12:** Quality of life; physical composite (related to daily and social activity); daily activity (such as work); mental composite (related to concentration in daily activity or work or mental healt interfering with social activities) **EQ-5D:** Quality of life (including usual activities)	6
					Standard care		NS				
Greenwood 2015 (21)	ExeRT, ISRCTN 43892586	Parallel RCT	UK	20	Tailored aerobic training (cycles, a treadmill) 3 days/week for 12 weeks	53.9±10.7	77.0	NS	SF-36	Quality of life; social functioning (including social activity and emotional problems interfered with normal social activities); mental composite (including emotional health problems related to work or other activities, role - emotion); physical composite (including	2.8
				20	High-intensity resistance training 3 days/week for 12 weeks	54.6±10.6	54.0	NS			
				20	No exercise	49.5±10.6	50.0	NS			

										limitation in activities, role - physical)	
Hedayati 2017 (22)	IRCT2015111625063N1	Parallel RCT	Iran	35	Expressive emotion education (based on feeling on kidney failure)	39.3±13.0	40.0	88.6	GHQ-28	Quality of life, social dysfunction	2.8
				35	Recording education (education based on the facts of kidney failure and not emotions)	40.8±14.4	45.7	97.1			
Henggeler 2018 (23)	INTENT, ACTRN12614000155695	Parallel RCT	New Zealand	19	Intensive nutrition (individualized nutrition and exercise counselling)	49.2±14.6	67	67.0	SF-36	Quality of life; social functioning (including social activity and emotional problems interfered with normal social activities); mental composite (including emotional health problems related to work or other activities, role - emotion); physical composite (including limitation in activities, role - physical)	12
				18	Standard nutrition (guideline based)	48.3±13.9	72	61.0			
Hu 2022 (24)	NS	Parallel RCT	China	30	Nursing intervention based on health belief model (manual and detailed training)	46.0±3.4	53.3	NS	Unspecified QoL scale assessing LP	Quality of life; social	6
				30	Routine nursing	45.9±3.6	60.0	NS			
Kastelz 2021 (25)	NCT02409901	Parallel RCT	US	93	Resistance-based	47.3±11.6	60.0	50.0	PROMIS Global	**PROMIS Global Health SF:** global physical	12

					exercise trainings				Health SF, PROMIS-29	health, global mental health **PROMIS-29:** satisfaction with social role	
				42	Standard care	43.2±12.0	45.5	33.3			
Lee 2000 (26)	NS	Parallel RCT	Taiwan	22	Pharmacy-based (CsA) TDM service	NS	52.3	NS	SF-36	Quality of life; social functioning (including social activity and emotional problems interfered with normal social activities); mental composite (including emotional health problems related to work or other activities, role - emotion); physical composite (including limitation in activities, role - physical)	6
				21	Non-pharmacist treatment	NS	58.8	NS			
Loftus-Farren 2012* (27)	NS	Parallel RCT	US	26	Mini incision	41.1±15.0	50.0	0	SF-36	Quality of life; social functioning (including social activity and emotional problems interfered with normal social activities); mental composite (including emotional health problems related to work or other activities, role - emotion); physical composite (including limitation in activities, role - physical)	12
				25	Gibson incision	47.7±14.2	72.0	0			
Mahrova 2012* (28)	NS	Parallel RCT	Czech Republic	21^	Exercise (joint mobility, muscle strength, nimbleness, dynamic stability, 3	59.1±10.8	NS	NS	KDQOL-SF	Quality of life (including work, sexual function)	12

					times/week for 6 months						
					Exercise + nutrition	57.0±8.4	NS	NS			
					Nutrition (substitution with keto-aminoacidic)	69.9±25.4	NS	NS			
					Control	65.1±17.8	NS	NS			
Marinho 2021 (29)	NCT03120377	Parallel RCT	Brazil	6	Whole-body vibration exercises (stretching) twice a week for 12 weeks	43.2, SD not reported	67.0	NS	SF-36	Quality of life; social functioning (including social activity and emotional problems interfered with normal social activities); mental composite (including emotional health problems related to work or other activities, role - emotion); physical composite (including limitation in activities, role - physical)	2.8
				6	Sham group	44.5, SD not reported	67.0	NS			
Ooms 2020 (30)	SPLINT	Parallel RCT, NL4358 (NTR4498)	The Netherlands	100	Suprapubic externalized single J stents	52.0±14.0	61.0	0	SF-36, EQ-5D	**SF-36:** Quality of life; social functioning (including social activity and emotional problems interfered with normal social activities); mental composite (including emotional health problems related to work or other activities, role - emotion); physical composite (including limitation in activities, role - physical)<br>**EQ-5D:** Quality of life (including usual activities)	12
				100	Suprapubic externalized without stent	55.0±13.0	63.0	0			

Riess 2014 (31)	NS	Parallel RCT	Canada	16	Aerobic exercise (cycle ergometer)	56.9±12.2	50.0	56.3	SF-36	Quality of life; social functioning (including social activity and emotional problems interfered with normal social activities); mental composite (including emotional health problems related to work or other activities, role - emotion); physical composite (including limitation in activities, role - physical)	2.8
				15	Standard care without exercise	52.4±14.3	40.0	60.0			
Tzvetanov 2014 (32)	NS	Parallel RCT	US	9	Resistance-based weight training with two 1-hour sessions/week (including physical fitness, psychology, and nutrition)	46.0±6.9	50.0	30.0	SF-36	Quality of life; social functioning (including social activity and emotional problems interfered with normal social activities); mental composite (including emotional health problems related to work or other activities, role - emotion); physical composite (including limitation in activities, role - physical)	12
				8	Standard care	45.0±19.0	37.5	50.0			
Yan 2021 (33)	NS	Parallel RCT	China	37	Nursing collaborative Care Model	43.2±12.6	62.2	32.4	QoL-RT	Quality of life, society	3
				36	Conventional nursing	44.5±12.8	69.2	36.1			

*Studies were available as abstract only; Data reported as mean with or without standard deviation (SD); ^Data were reported on the overall population; !Full data available on clinicaltrial.gov; PROM, Patient Reported Outcome Measure; M, male; NS, Not stated; UK, United Kingdom; US, United States; IV, intravenous; ESA, Erythropoietin stimulating agents; Hb, Hemoglobin; TDM, Therapeutic drug monitoring; CsA, cyclosporine A; MMF, Mycophenolate mofetil; MPS, Mycophenolate Sodium; EC-MPS, Enteric-Coated Mycophenolate Sodium; Tac, Tacrolimus; SRL, Sirolimus; CNI, Calcineurin Inhibitor; AZA, Azathioprine; PUFA, Polyunsaturated Fatty Acids; ATG, Antithymocyte Globulin; r-ATG, Rabbit Antithymocyte Globulin; IL-2 RA, Interleukin-2 Receptor Antagonist; SF-12, 12-Item Short Form Health Survey; SF-36, 36-Item Short Form Health Survey; EQ-5D, EuroQol-5D; GIQLI, Gastrointestinal Quality of Life Index (GIQLI); PGWBI, Psychological General Wellbeing Index; KDQOL-SF, Kidney Disease Quality of Life-Short Form; GHQ-28, General Health Questionnaire-28; PROMIS Global Health SF, Patient-Reported Outcomes Measurement Information System Global Health Short Form; PROMIS-29, Patient-Reported Outcomes Measurement Information System 29 Items; QoL-RT, Quality of Life Renal Transplant Recipients; QoL, quality of life.

Table S3. List of included interventions

- Angiotensin-converting enzyme inhibitors (ACEi) versus calcium channel blockers (CCB)
    - Lisinopril versus nifedipine (one study)
- Prebiotics versus placebo/control
    - Marine n-3 polyunsaturated fatty acids (PUFA) versus olive oil (one study)
    - Banana-resistant starch versus placebo (one study)
- Erythropoietin-stimulating agents (ESA) versus no treatment
    - Epoetin beta versus no treatment (one study)
- ESA in different target hemoglobin (Hb) regimen
    - ESA (target Hb 11-12 g/dL) versus ESA (target Hb 13-15 g/dL) (one study)
- Immunosuppressive therapy versus another immunosuppressive therapy
    - Cyclosporin A (CsA) versus fusion protein (belatacept) at less or more intense administration (two studies)
    - Standard-dose CsA plus mycophenolate mofetil (MMF) plus corticosteroids versus daclizumab induction plus MMF plus corticosteroid with a low-dose of CsA (one study)
    - Standard-dose CsA plus MMF plus corticosteroids versus low-dose tacrolimus (Tac) (one study)
    - Standard-dose CsA plus MMF plus corticosteroids versus low-dose sirolimus (SRL)
    - Daclizumab induction plus MMF plus corticosteroid with a low-dose of CsA versus low-dose Tac (one study)

    - Daclizumab induction plus MMF plus corticosteroid with a low-dose of CsA versus low-dose SRL (one study)
    - Low-dose Tac versus low-dose SRL (one study)
    - SRL plus corticosteroids versus SRL plus CsA plus corticosteroids (one study)
    - Enteric-coated mycophenolate sodium (EC-MPS) versus MMF (two studies)
    - CsA versus Tac (two studies)
    - Azathioprine versus MMF (one study)
- Immunosuppressive therapy versus non-steroidal treatment
    - Prednisolone plus induction therapy of interleukin-2 (IL-2) versus non-steroidal treatment + induction therapy of IL-2 (one study)
- Thymoglobulin versus other immunosuppressive treatment/induction therapy
    - Rabbit antithymocyte globulin (r-ATG) versus induction therapy of interleukin-2 receptor antagonist (IL-2 RA) daclizumab (one study)
    - r-ATG versus alemtuzumab (one study)
    - r-ATG plus belatacept versus alemtuzumab plus belatacept (one study)
    - r-ATG plus Tac versus alemtuzumab plus belatacept (one study)
    - r-ATG plus belatacept versus r-ATG plus Tac (one study)
- Exercise versus standard care/no treatment
    - Aerobic exercise versus no exercise (two studies)
    - Aerobic exercise versus control (no clear definition was provided) (one study)
    - Aerobic exercise plus nutrition versus control (no clear definition was provided) (one study)
    - Resistance training versus no exercise (one study)
    - Resistance based exercise training versus standard care (one study)
    - Resistance based weight training versus standard care (one study)

  - Whole-body vibrating exercise (stretching) versus sham group (one study)
- Exercise versus another exercise
  - Aerobic exercise versus resistance training (one study)
- Exercise versus nutrition
  - Aerobic exercise training versus nutrition alone (one study)
  - Aerobic exercise training plus nutrition versus nutrition alone (one study)
- Exercise versus exercise plus nutrition
  - Aerobic exercise training versus aerobic exercise training plus nutrition (one study)
- Nutrition vs control
  - Intensive nutrition (individualized nutrition and exercise counselling) versus standard nutrition (two studies)
  - Nutrition alone versus control (no clear definition was provided) (one study)
- Education versus standard care
  - Behavioural contact intervention versus standard care (one study)
  - Nursing intervention based on belief model (providing manual and detailed training or care model) versus standard nursing (two studies)
  - Pharmacy-based cyclosporin therapeutic drug monitoring service versus non-pharmacist treatment (one study)
- Education versus education
  - Expressive emotional education versus recording education (one study)
- Surgical incision versus another surgical incision
  - Mini incision versus Gibson incision (one study)
  - Stent (suprapubic externalized single J stent) versus no-stent (suprapubic externalized without stent) ureteroneocystostomy (one study)

- Calcium-channel blockers (CCB) versus placebo
    - Diltiazem (one study)

Table S4. Risk of bias summary

Study ID	Sequence generation	Allocation concealment	Blinding of participants and investigators	Blinding of outcome assessment	Incomplete outcome data	Selective reporting	Other bias
Aasebo 2005	Unclear risk	Unclear risk	Low risk	Unclear risk	High risk	High risk	Unclear risk
Aasebo 2019	Low risk	Unclear risk	Low risk	Unclear risk	Unclear risk	High risk	Low risk
Al-Otaibi 2019	Unclear risk	Unclear risk	High risk	Unclear risk	High risk	High risk	Unclear risk
Anderson 2011	Unclear risk	Unclear risk	High risk	Unclear risk	High risk	High risk	Unclear risk
Baltar 2002	Unclear risk	Unclear risk	High risk	Unclear risk	High risk	High risk	High risk
Chan 2022	Low risk	Low risk	Low risk	Unclear risk	Unclear risk	Low risk	Low risk
Chisholm-Burns 2011	Unclear risk	Unclear risk	High risk	Unclear risk	High risk	High risk	Unclear risk
Durrbach 2010	Low risk	Low risk	High risk	Unclear risk	Unclear risk	Low risk	High risk
Greenwood 2015	Low risk	Unclear risk	High risk	Unclear risk	High risk	Low risk	Low risk
Hedayati 2017	Low risk	Unclear risk	Low risk	Unclear risk	Unclear risk	High risk	Unclear risk
Henggeler 2018	Low risk	Low risk	High risk	Low risk	High risk	High risk	Low risk
Hu 2022	Unclear risk	Unclear risk	High risk	Unclear risk	Low risk	High risk	Unclear risk
Kastelz 2021	Low risk	Unclear risk	High risk	Unclear risk	High risk	High risk	Low risk
Kumana 2003	Unclear risk	Unclear risk	Low risk	Unclear risk	Low risk	Low risk	Low risk
Lee 2000	Unclear risk	Unclear risk	High risk	Unclear risk	High risk	High risk	Unclear risk
Loftus-Farren 2012	Unclear risk	Unclear risk	High risk	Unclear risk	High risk	High risk	Unclear risk
Mahrova 2012	Unclear risk	Unclear risk	High risk	Unclear risk	High risk	High risk	Unclear risk
Marinho 2021	Low risk	Low risk	Low risk	Unclear risk	Unclear risk	High risk	Low risk
Ooms 2020	Unclear risk	Low risk	High risk	Unclear risk	Unclear risk	High risk	Low risk
Oppenheimer 2009	Unclear risk	Unclear risk	High risk	Unclear risk	High risk	High risk	Unclear risk
Ortega 2011	Unclear risk	Unclear risk	High risk	Unclear risk	Unclear risk	High risk	High risk
Painter 2003	Unclear risk	Unclear risk	High risk	Unclear risk	High risk	High risk	High risk
Palanisamy 2015	Unclear risk	Unclear risk	High risk	Unclear risk	High risk	High risk	Low risk
Pile 2020	Low risk	Unclear risk	High risk	Unclear risk	Unclear risk	High risk	Low risk
Riess 2014	Low risk	Low risk	High risk	Unclear risk	Unclear risk	High risk	Low risk
Rohan 2019	Unclear risk	Unclear risk	High risk	Unclear risk	High risk	High risk	Unclear risk
Russ 2007	Unclear risk	Unclear risk	High risk	Unclear risk	High risk	High risk	Low risk
Shehata 2009	Unclear risk	Unclear risk	High risk	Unclear risk	Unclear risk	High risk	High risk
Shield 1997	Unclear risk	Unclear risk	High risk	Unclear risk	High risk	High risk	Unclear risk
Tzvetanov 2014	Unclear risk	Unclear risk	High risk	Unclear risk	Low risk	High risk	Unclear risk
Vincenti 2010	Low risk	Low risk	High risk	Unclear risk	Unclear risk	Low risk	High risk
Walker 2007	Unclear risk	Unclear risk	High risk	Unclear risk	Unclear risk	High risk	High risk
Yan 2021	Unclear risk	Unclear risk	High risk	Unclear risk	Low risk	High risk	Unclear risk

Figure S1.1. Prebiotics vs placebo/control: Change in quality of life

	Prebiotics			Placebo/control			Mean Difference
Study or Subgroup	Mean	SD	Total	Mean	SD	Total	IV, Random, 95% CI
Chan 2022	-0.2	0.2	27	-0.3	0.2	29	0.10 [-0.00, 0.20]

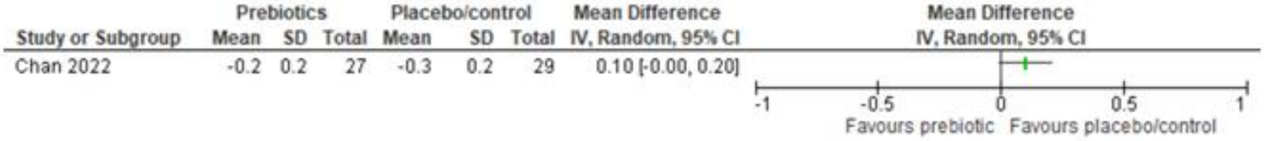

Figure S2.1. ESA vs no treatment: Change in quality of life

	ESA			No treatment			Mean Difference
Study or Subgroup	Mean	SD	Total	Mean	SD	Total	IV, Random, 95% CI
Pile 2020	2.8	23	28	6.6	2.3	27	-3.80 [-12.36, 4.76]

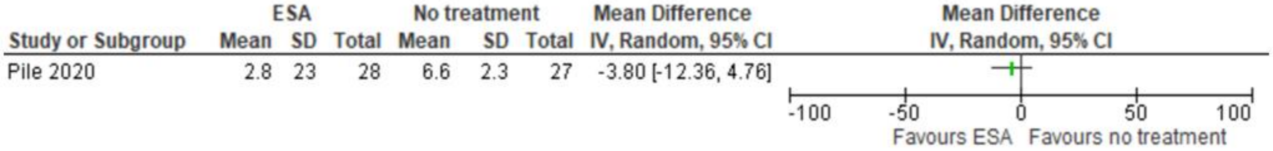

Figure S3.1. Cyclosporin vs fusion protein (belatacept): Social functioning

	Cyclosporine			Belatacept			Mean Difference
Study or Subgroup	Mean	SD	Total	Mean	SD	Total	IV, Random, 95% CI
Vincenti 2010	46.6	10.68	204	47.6	3.23	425	-1.00 [-2.50, 0.50]

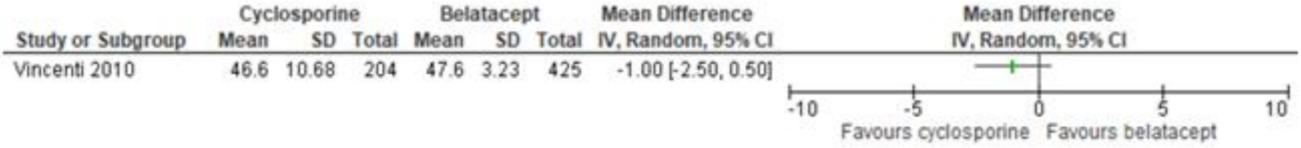

Figure S3.2. Cyclosporin vs fusion protein (belatacept): Change in social functioning

	Cyclosporine			Belatacept				Mean Difference
Study or Subgroup	Mean	SD	Total	Mean	SD	Total	Weight	IV, Random, 95% CI
Durrbach 2010	1.4	10.238	154	3.8	3.2	298	44.8%	-2.40 [-4.06, -0.74]
Vincenti 2010	5.1	10.1983	192	6.3	3	404	55.2%	-1.20 [-2.67, 0.27]
**Total (95% CI)**			**346**			**702**	**100.0%**	**-1.74 [-2.91, -0.57]**

Heterogeneity: $Tau^2 = 0.08$; $Chi^2 = 1.13$, $df = 1$ ($P = 0.29$); $I^2 = 11\%$
Test for overall effect: $Z = 2.91$ ($P = 0.004$)

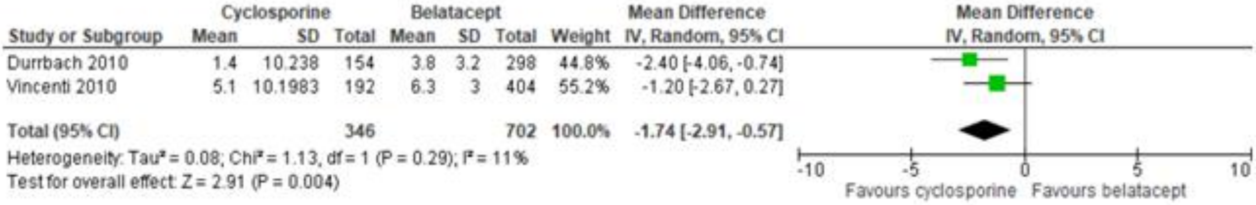

Figure 3.3. Cyclosporin vs fusion protein (belatacept): Mental functioning

	Cyclosporine			Belatacept			Mean Difference
Study or Subgroup	Mean	SD	Total	Mean	SD	Total	IV, Random, 95% CI
Vincenti 2010	46.9	11.6	203	48.5	3.4	419	-1.60 [-3.23, 0.03]

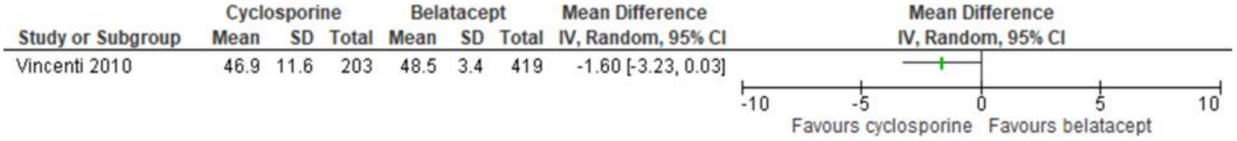

Figure S3.4. Cyclosporin vs fusion protein (belatacept): Change in mental functioning

	Cyclosporine			Belatacept			Mean Difference
Study or Subgroup	Mean	SD	Total	Mean	SD	Total	IV, Random, 95% CI
Vincenti 2010	2.6	0.756	190	4.8	0.9	394	-2.20 [-2.34, -2.06]

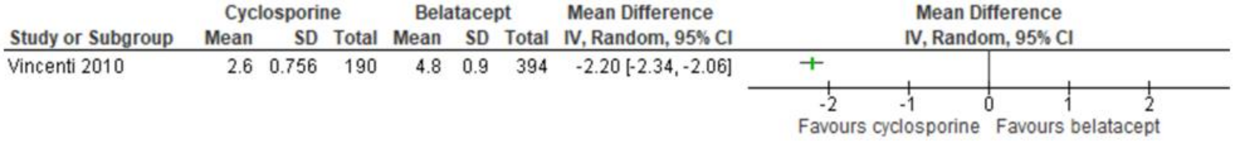

Figure S3.5. Cyclosporin vs fusion protein (belatacept): Physical functioning

	Cyclosporine			Belatacept			Mean Difference
Study or Subgroup	Mean	SD	Total	Mean	SD	Total	IV, Random, 95% CI
Vincenti 2010	47.1	9.47	203	49	3	419	-1.90 [-3.23, -0.57]

Mean Difference IV, Random, 95% CI

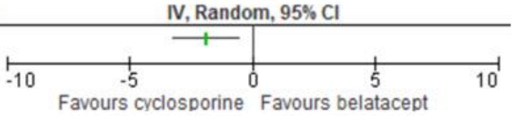

Figure S3.6. Cyclosporin vs fusion protein (belatacept): Change in physical functioning

	Cyclosporine			Belatacept			Mean Difference
Study or Subgroup	Mean	SD	Total	Mean	SD	Total	IV, Random, 95% CI
Vincenti 2010	4.9	0.633	190	6.31	0.79	394	-1.41 [-1.53, -1.29]

Mean Difference IV, Random, 95% CI

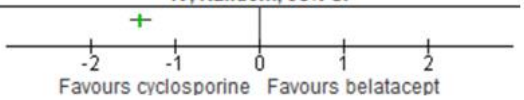

Figure S4.1. Standard-dose cyclosporin vs low- or standard-dose tacrolimus: Social functioning

	Standard-dose CsA			Low-dose Tac			Mean Difference
Study or Subgroup	Mean	SD	Total	Mean	SD	Total	IV, Random, 95% CI
Walker 2007	77.5	22.7	10	86.4	15.3	11	-8.90 [-25.62, 7.82]

Mean Difference IV, Random, 95% CI

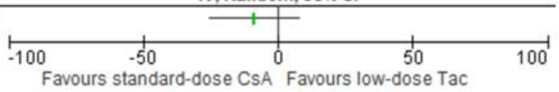

Figure S4.2. Standard-dose cyclosporin vs low- or standard-dose tacrolimus: Role – emotional

	Standard-dose CsA			Low-dose Tac			Mean Difference
Study or Subgroup	Mean	SD	Total	Mean	SD	Total	IV, Random, 95% CI
Walker 2007	55.6	52.7	9	84.8	27.3	11	-29.20 [-67.22, 8.82]

Mean Difference IV, Random, 95% CI

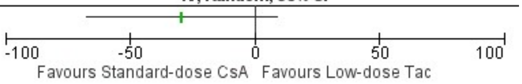

Figure S4.3. Standard-dose cyclosporin vs low- or standard-dose tacrolimus: Role – physical

	Standard-dose CsA			Low-dose Tac			Mean Difference
Study or Subgroup	Mean	SD	Total	Mean	SD	Total	IV, Random, 95% CI
Walker 2007	56.3	49.6	8	87.5	24.3	10	-31.20 [-68.73, 6.33]

Mean Difference IV, Random, 95% CI

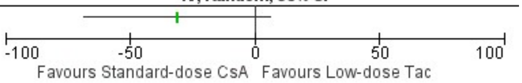

Figure S5.1. Cyclosporine plus sirolimus and steroids vs sirolimus plus steroids: Social functioning

	Cyclosporine + sirolimus + steroids			Sirolimus + steroids			Mean Difference
Study or Subgroup	Mean	SD	Total	Mean	SD	Total	IV, Random, 95% CI
Russ 2007	79.1	29.319	176	85.6	26.3691	181	-6.50 [-12.29, -0.71]

Mean Difference IV, Random, 95% CI

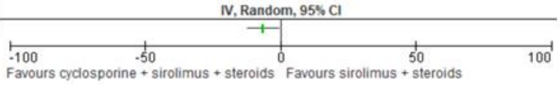

Figure S5.2. Cyclosporine plus sirolimus and steroids vs sirolimus plus steroids: Role-emotional

	Cyclosporine + sirolimus + steroids			Sirolimus + steroids			Mean Difference
Study or Subgroup	Mean	SD	Total	Mean	SD	Total	IV, Random, 95% CI
Russ 2007	81.6	44.8529	172	84.4	42.1275	180	-2.80 [-11.90, 6.30]

Mean Difference IV, Random, 95% CI

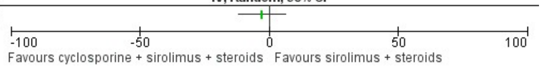

Figure S5.3. Cyclosporine plus sirolimus and steroids vs sirolimus plus steroids: Role - physical

	Cyclosporine + sirolimus + steroids			Sirolimus + steroids			Mean Difference
Study or Subgroup	Mean	SD	Total	Mean	SD	Total	IV, Random, 95% CI
Russ 2007	66.9	54	174	74.8	46.3	179	-7.90 [-18.41, 2.61]

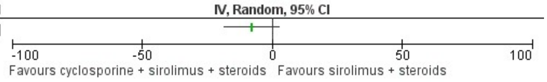

Figure S6.1. Enteric-coated mycophenolate sodium vs mycophenolate mofetil: Quality of life

	EC-MPS			MMF			Mean Difference
Study or Subgroup	Mean	SD	Total	Mean	SD	Total	IV, Random, 95% CI
Ortega 2011	73.4	18.5	56	66.9	21.1	51	6.50 [-1.05, 14.05]

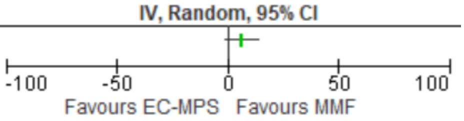

Figure S6.2. Enteric-coated mycophenolate sodium vs mycophenolate mofetil: Change in physical functioning

	EC-MPS			MMF			Mean Difference
Study or Subgroup	Mean	SD	Total	Mean	SD	Total	IV, Random, 95% CI
Shehata 2009	2.7	15.6678	68	0.1	14.8395	61	2.60 [-2.67, 7.87]

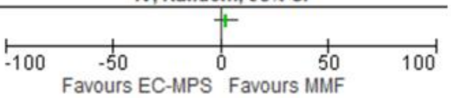

Figure S7.1. Thymoglobulin vs IL-2 RA: Decrease in social functioning

	Thymoglobulin		IL-2 RA		Risk Ratio
Study or Subgroup	Events	Total	Events	Total	M-H, Random, 95% CI
Palanisamy 2015	10	63	17	48	0.45 [0.23, 0.89]

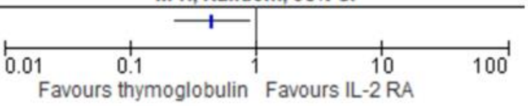

Figure S7.2. Thymoglobulin vs IL-2 RA: Role – emotional

	Thymoglobulin		IL-2 RA		Risk Ratio
Study or Subgroup	Events	Total	Events	Total	M-H, Random, 95% CI
Palanisamy 2015	16	63	23	48	0.53 [0.32, 0.89]

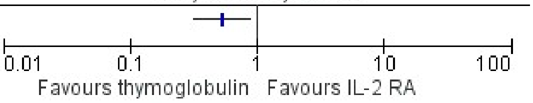

Figure S7.3. Thymoglobulin vs IL-2 RA: Role - physical

	Thymoglobulin		IL-2 RA		Risk Ratio
Study or Subgroup	Events	Total	Events	Total	M-H, Random, 95% CI
Palanisamy 2015	32	63	23	48	1.06 [0.72, 1.55]

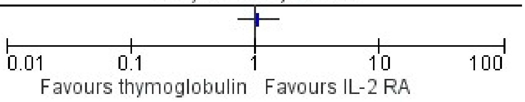

Figure S8.1. Exercise vs standard care: Change in social functioning

	Exercise			Standard care			Mean Difference
Study or Subgroup	Mean	SD	Total	Mean	SD	Total	IV, Random, 95% CI
Riess 2014	15.2	26.5	16	-9.82	17.11	15	25.02 [9.41, 40.63]

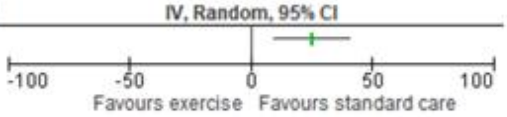

Figure S8.2. Exercise vs standard care: Change in overall quality of life

	Exercise			Standard care			Mean Difference
Study or Subgroup	Mean	SD	Total	Mean	SD	Total	IV, Random, 95% CI
Riess 2014	8.6	13.4	16	-3.4	12.1	15	12.00 [3.02, 20.98]

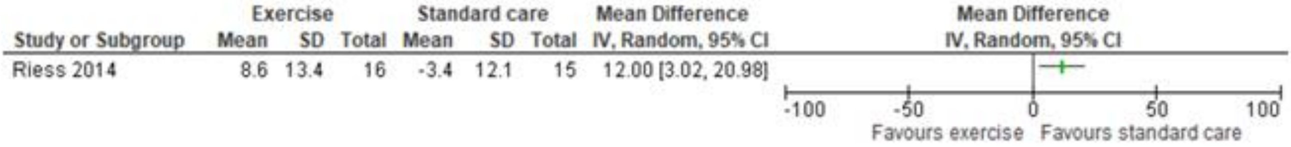

Figure S8.3. Exercise vs standard care: Mental functioning

	Exercise			Standard care			Mean Difference
Study or Subgroup	Mean	SD	Total	Mean	SD	Total	IV, Random, 95% CI
Greenwood 2015	52.2	13.5	13	55.7	5.1	20	-3.50 [-11.17, 4.17]

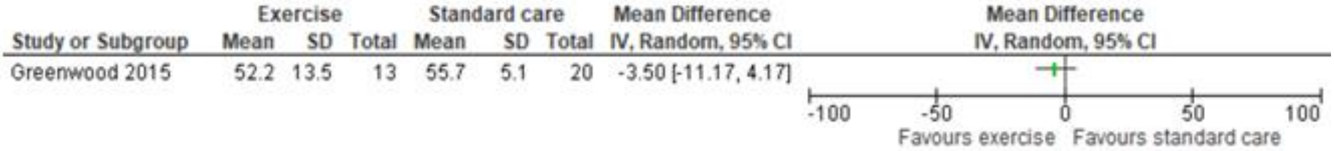

Figure S8.4. Exercise vs standard care: Change in mental functioning

	Exercise			Standard care			Mean Difference
Study or Subgroup	Mean	SD	Total	Mean	SD	Total	IV, Random, 95% CI
Riess 2014	-6.9	42.1	16	-9.4	23.9	15	2.50 [-21.41, 26.41]

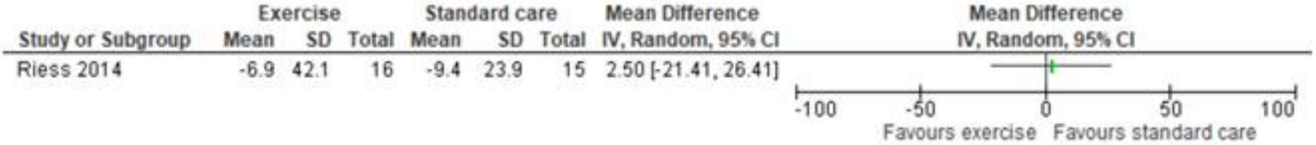

Figure S8.5. Exercise vs standard care: Physical functioning

	Exercise			Standard care			Mean Difference
Study or Subgroup	Mean	SD	Total	Mean	SD	Total	IV, Random, 95% CI
Greenwood 2015	37.8	7.2	13	41.3	8.3	20	-3.50 [-8.84, 1.84]

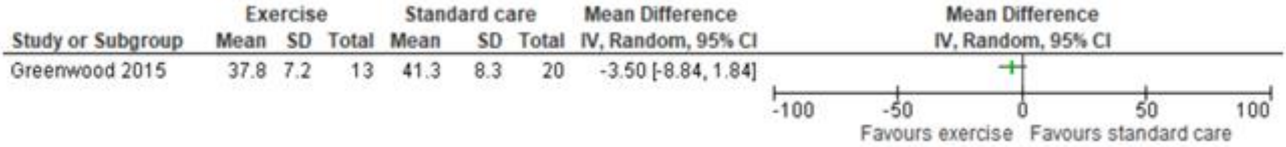

Figure S9.1. Aerobic exercise vs resistance training: Mental functioning

	Aerobic			Resistance			Mean Difference
Study or Subgroup	Mean	SD	Total	Mean	SD	Total	IV, Random, 95% CI
Greenwood 2015	52.2	13.5	13	51.5	14.9	13	0.70 [-10.23, 11.63]

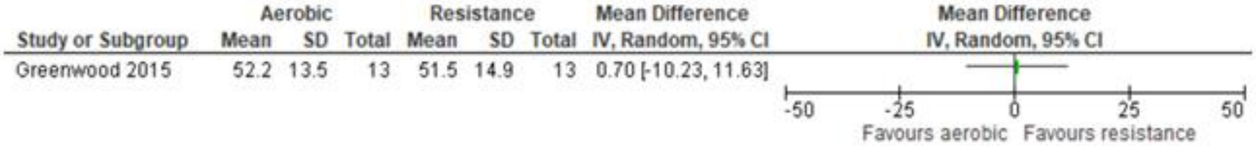

Figure S9.2. Aerobic exercise vs resistance training: Physical functioning

	Aerobic			Resistance			Mean Difference
Study or Subgroup	Mean	SD	Total	Mean	SD	Total	IV, Random, 95% CI
Greenwood 2015	37.8	7.2	13	43.5	19.9	13	-5.70 [-17.20, 5.80]

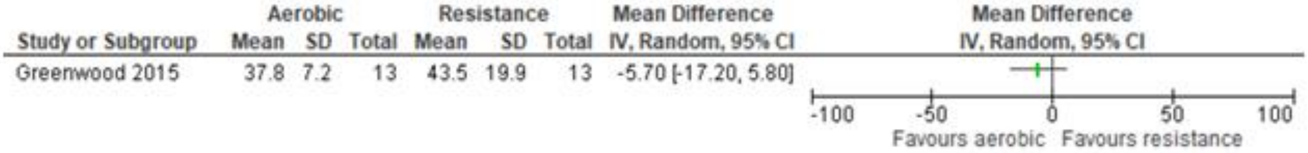

Figure S10.1. Resistance training vs standard care: Satisfaction with social role

	Resistance training			Standard care			Mean Difference
Study or Subgroup	Mean	SD	Total	Mean	SD	Total	IV, Random, 95% CI
Kastelz 2021	54.03	9.44	52	52.82	8.81	28	1.21 [-2.94, 5.36]

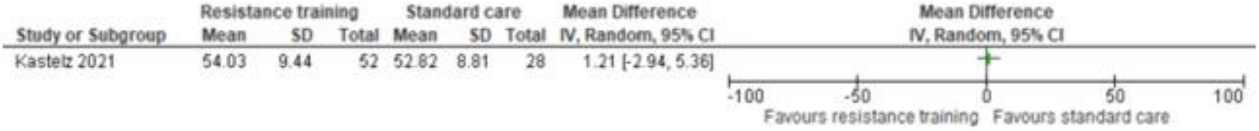

Figure S10.2. Resistance training vs standard care: Quality of life

Study or Subgroup	Resistance training Mean	SD	Total	Standard care Mean	SD	Total	Mean Difference IV, Random, 95% CI
Tzvetanov 2014	58.3	13	9	43.6	22	8	14.70 [-2.75, 32.15]

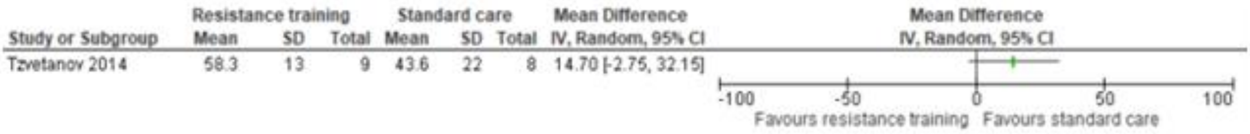

Figure S10.3. Resistance training vs standard care: Mental functioning

Study or Subgroup	Resistance training Mean	SD	Total	Standard care Mean	SD	Total	Weight	Std. Mean Difference IV, Random, 95% CI
Greenwood 2015	51.5	14.9	13	55.7	5.1	20	45.2%	-0.41 [-1.11, 0.30]
Kastelz 2021	53.11	7.95	52	49.53	6.69	28	54.8%	0.47 [0.00, 0.94]
**Total (95% CI)**			**65**			**48**	**100.0%**	**0.07 [-0.78, 0.93]**

Heterogeneity: Tau² = 0.29; Chi² = 4.13, df = 1 (P = 0.04); I² = 76%
Test for overall effect: Z = 0.17 (P = 0.87)

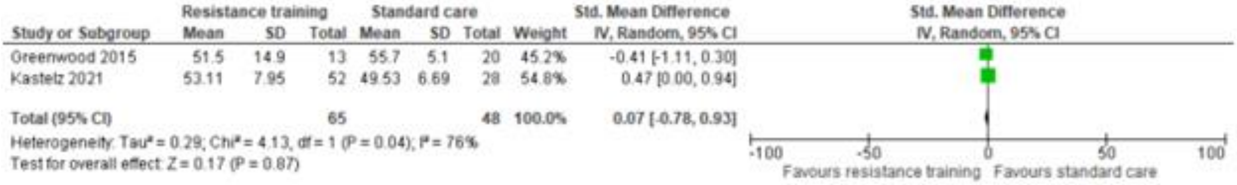

Figure S10.4. Resistance training vs standard care: Physical functioning

Study or Subgroup	Resistance training Mean	SD	Total	Standard care Mean	SD	Total	Weight	Std. Mean Difference IV, Random, 95% CI
Greenwood 2015	43.5	19.9	13	41.3	8.3	20	32.1%	0.15 [-0.55, 0.85]
Kastelz 2021	51.58	8.3	52	46.6	8.24	28	67.9%	0.60 [0.13, 1.06]
**Total (95% CI)**			**65**			**48**	**100.0%**	**0.45 [0.05, 0.86]**

Heterogeneity: Tau² = 0.01; Chi² = 1.06, df = 1 (P = 0.30); I² = 6%
Test for overall effect: Z = 2.20 (P = 0.03)

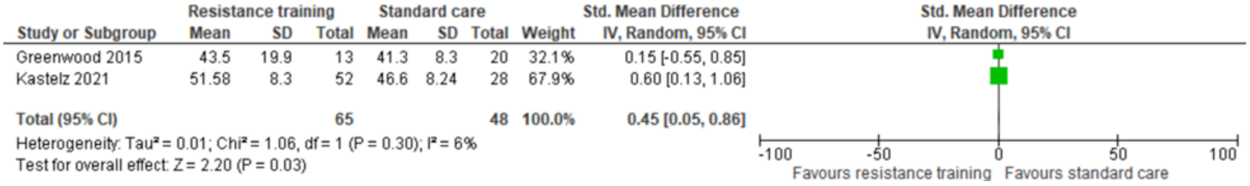

Figure S11.1. Intense nutrition vs standard nutrition: Social functioning

Study or Subgroup	Intense nutrition Mean	SD	Total	Standard nutrition Mean	SD	Total	Mean Difference IV, Random, 95% CI
Henggeler 2018	91.7	18.6676	18	92.3	14.0007	18	-0.60 [-11.38, 10.18]

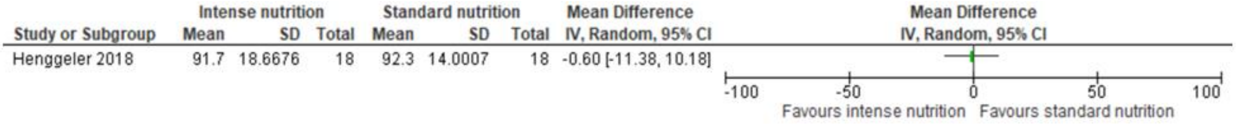

Figure S11.2. Intense nutrition vs standard nutrition: Role - emotional

Study or Subgroup	Intense nutrition Mean	SD	Total	Standard nutrition Mean	SD	Total	Mean Difference IV, Random, 95% CI
Henggeler 2018	93.1	18.7	18	94.9	11.9	18	-1.80 [-12.04, 8.44]

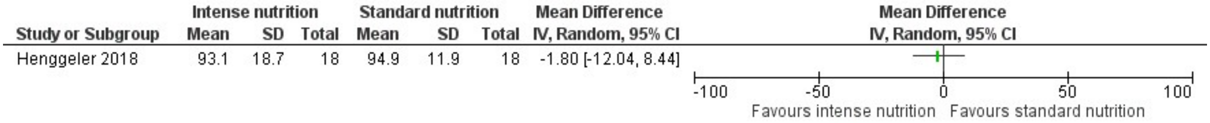

Figure S11.3. Intense nutrition vs standard nutrition: Role - physical

Study or Subgroup	Intense nutrition Mean	SD	Total	Standard nutrition Mean	SD	Total	Mean Difference IV, Random, 95% CI
Henggeler 2018	78.8	31	18	92.8	10.6	18	-14.00 [-29.14, 1.14]

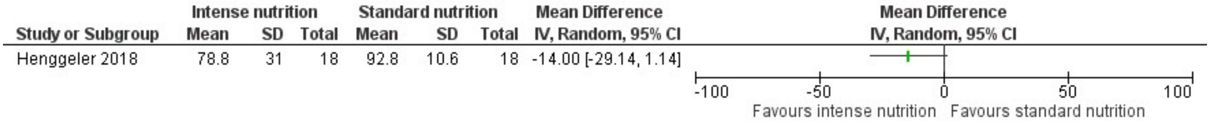

Figure S12.1. Expressive emotion education vs recording education without emotion disclosure: Social dysfunction

Study or Subgroup	Expressive emotion Mean	SD	Total	Recording education Mean	SD	Total	Mean Difference IV, Random, 95% CI
Hedayati 2017	1.24	1.07	34	3.2	1.47	30	-1.96 [-2.60, -1.32]

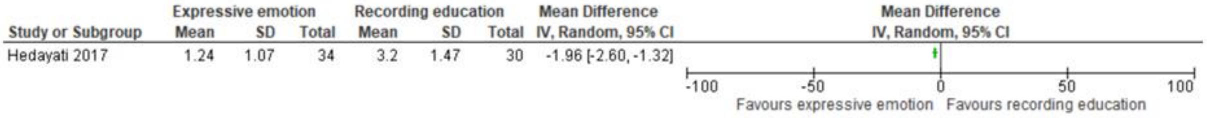

Figure S12.2. Expressive emotion education vs recording education without emotion disclosure: Quality of life

	Expressive emotion			Recording education			Mean Difference
Study or Subgroup	Mean	SD	Total	Mean	SD	Total	IV, Random, 95% CI
Hedayati 2017	8.09	4.85	34	14.53	4.73	30	-6.44 [-8.79, -4.09]

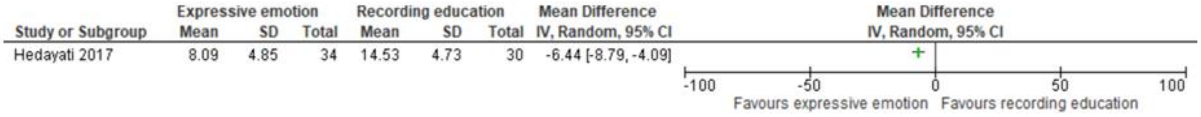

Figure S13.1. Education vs standard care: Social functioning

	Education			Standard care			Mean Difference
Study or Subgroup	Mean	SD	Total	Mean	SD	Total	IV, Random, 95% CI
Hu 2022	12.12	3.77	30	16.55	2.77	30	-4.43 [-6.10, -2.76]

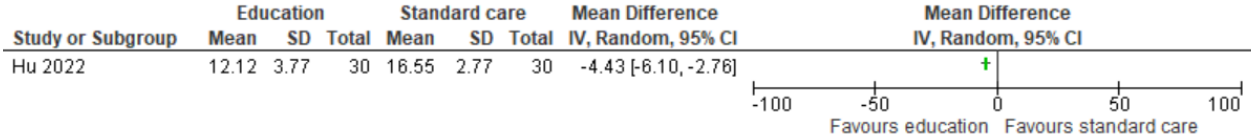

Figure S14.1. Steroid versus no steroid treatment: Role – emotional

	Steroid			Non-steroidal			Mean Difference
Study or Subgroup	Mean	SD	Total	Mean	SD	Total	IV, Random, 95% CI
Painter 2003	77.7	37.3	14	91.7	23.6	9	-14.00 [-38.89, 10.89]

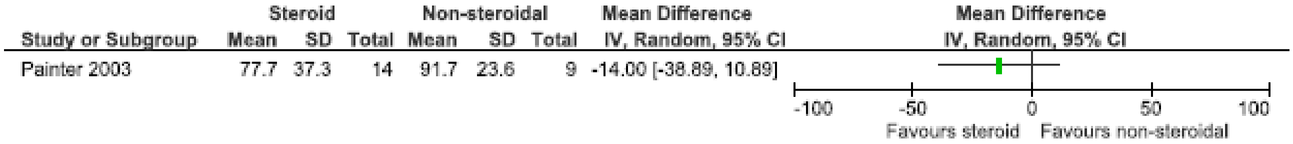

Figure S14.2. Steroid versus no steroid treatment: Mental functioning

	Steroid			Non-steroidal			Mean Difference
Study or Subgroup	Mean	SD	Total	Mean	SD	Total	IV, Random, 95% CI
Painter 2003	52.3	9.1	14	56	3.4	9	-3.70 [-8.96, 1.56]

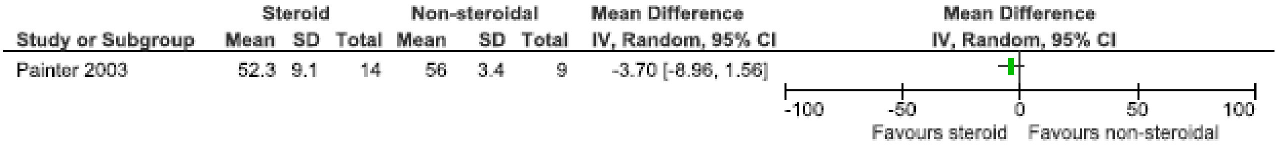

Figure S14.3. Steroid versus no steroid treatment: Role – physical

	Steroid			Non-steroidal			Mean Difference
Study or Subgroup	Mean	SD	Total	Mean	SD	Total	IV, Random, 95% CI
Painter 2003	63.9	43.5	14	75	40	9	-11.10 [-45.77, 23.57]

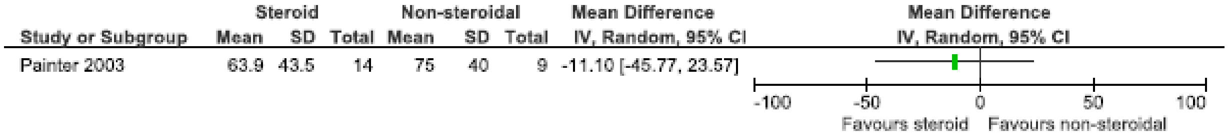

Figure S14.4. Steroid versus no steroid treatment: Physical functioning

	Steroid			Non-steroidal			Mean Difference
Study or Subgroup	Mean	SD	Total	Mean	SD	Total	IV, Random, 95% CI
Painter 2003	42.6	12.1	14	48.4	8.7	9	-5.80 [-14.31, 2.71]

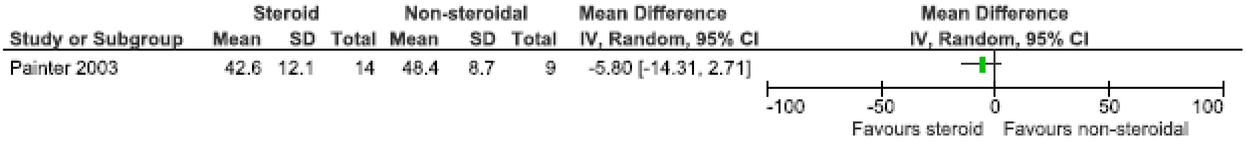

Figure S14.5. Steroid versus no steroid treatment: Social functioning

	Steroid			Non-steroidal			Mean Difference
Study or Subgroup	Mean	SD	Total	Mean	SD	Total	IV, Random, 95% CI
Painter 2003	68	25.1	14	84.4	17.4	9	-16.40 [-33.78, 0.98]

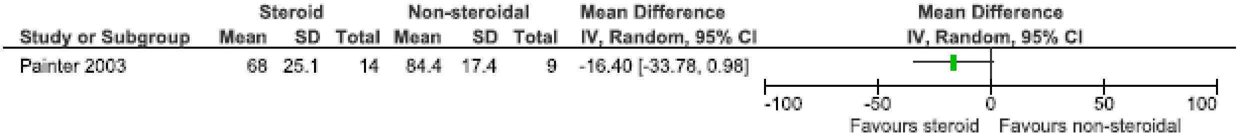

Supplementary References

1. Aasebo W, Midtvedt K, Hartmann A, Stavem K. Predictors of health-related quality of life in hypertensive recipients following renal transplantation. *Clin Transplant*. 2005;19(6):756-62.

2. Al-Otaibi T, Halim MA, Gheith O, Najeeb AM, Abo-Atteya H, Mahmoud T, et al. Impact of full correction of post-transplant anemia on cardiovascular system and quality of life in renal transplant recipients receiving erythropoietin stimulating agents: Prospective randomized controlled trial. *Transpl Int*. 2019;32(Supplement 2):396.

3. Anderson KT, Hart L, Hairston G, McPherson G, Stratta R, Rogers J, et al. Simultaneous kidney pancreas transplant recipients have similar perceived physical health quality of life as non-diabetic kidney transplant recipients at 1 and 2 years. *Am J Transplant*. 2011;2:434-5.

4. Baltar J, Ortega F, Rebollo P, Gomez E, Laures A, Alvarez-Grande J. [Changes in health-related quality of life in the first year of kidney transplantation]. *Nefrologia*. 2002;22(3):262-8.

5. Durrbach A, Pestana J, Pearson T, Vincenti F, Garcia VD, Campistol J, et al. A phase III study of belatacept versus cyclosporine in kidney transplants from extended criteria donors (BENEFIT-EXT study). *Am J Transplant*. 2010;10(3):547-57.

6. Kumana CR, Tong MK, Li CS, Lauder IJ, Lee JS, Kou M, et al. Diltiazem co-treatment in renal transplant patients receiving microemulsion cyclosporin. *Br J Clin Pharmacol*. 2003;56(6):670-8.

7. Oppenheimer F, Rebollo P, Grinyo JM, Ortega F, Sanchez-Plumed J, Gonzalez-Molina M, et al. Health-related quality of life of patients receiving low-toxicity immunosuppressive regimens: a substudy of the Symphony Study. *Transplantation*. 2009;87(8):1210-3.

8. Ortega F, Sanchez-Fructuoso A, Cruzado JM, Gomez-Alamillo JC, Alarcon A, Pallardo L, et al. Gastrointestinal quality of life improvement of renal transplant recipients converted from mycophenolate mofetil to enteric-coated mycophenolate sodium drugs or agents: mycophenolate mofetil and enteric-coated mycophenolate sodium. *Transplantation*. 2011;92(4):426-32.

9. Painter PL, Topp KS, Krasnoff JB, Adey D, Strasner A, Tomlanovich S, et al. Health-related fitness and quality of life following steroid withdrawal in renal transplant recipients. *Kidney Int*. 2003;63(6):2309-16.

10. Palanisamy AP, Al Manasra AR, Pilch NA, Dowden JE, Nadig SN, McGillicuddy JW, et al. Induction therapy: Clinical and quality of life outcomes in aged renal transplant recipients. *Clin Transplant*. 2015;29(3):222-6.

11. Pile T, Raftery M, Thuraisingham R, Kirwan CJ, Harwood S, Yaqoob MM. Treating posttransplant anemia with erythropoietin improves quality of life but does not affect progression of chronic kidney disease. *Exp Clin Transplant*. 2020;18(1):27-33.

12. Rohan JM, Leone JP, Woodle ES, Kaufman D, Shields AR, Wiseman A, et al. Patient-reported outcomes in a prospective multicenter trial of belatacept-based CNI-and corticosteroid-free immunosuppression regimens in kidney transplantation. *Am J Transplant*. 2019;19(Suppl 3):418-9.

13. Russ G, Jamieson N, Oberbauer R, Arias M, Murgia MG, Blancho G, et al. Three-year health-related quality-of-life outcomes for sirolimus-treated kidney transplant patients after elimination of cyclosporine. *Transpl Int*. 2007;20(10):875-83.

14. Shehata M, Bhandari S, Venkat-Raman G, Moore R, D'Souza R, Riad H, et al. Effect of conversion from mycophenolate mofetil to enteric-coated mycophenolate sodium on maximum tolerated dose and gastrointestinal symptoms following kidney transplantation. *Transpl Int*. 2009;22(8):821-30.

15. Shield CF, McGrath MM, Goss TF. Assessment of health-related quality of life in kidney transplant patients receiving tacrolimus (FK506)-based versus cyclosporine-based immunosuppression. FK506 Kidney Transplant Study Group. *Transplantation*. 1997;64(12):1738-43.

16. Vincenti F, Larsen C, Alberu J, Bresnahan B, Garcia VD, Kothari J, et al. Three-year outcomes from BENEFIT, a randomized, active-controlled, parallel-group study in adult kidney transplant recipients. *Am J Transplant*. 2012;12(1):210-7.

17. Walker RG, Cottrell S, Sharp K, Tripodi R, Nicholls KM, Fraser I, et al. Conversion of cyclosporine to tacrolimus in stable renal allograft recipients: Quantification of effects on the severity of gingival enlargement and hirsutism and patient-reported outcomes. *Nephrology*. 2007;12(6):607-14.

18. Aasebo W, Svensson M, Jenssen T, Eide IA. Marine n-3 Polyunsaturated Fatty Acid Supplementation and Quality of Life After Kidney Transplant. *Transplant Proc*. 2019;51(2):466-9.

19. Chan S, Hawley CM, Pascoe EM, Cao C, Campbell SB, Campbell KL, et al. Prebiotic Supplementation in Kidney Transplant Recipients for Preventing Infections and Gastrointestinal Upset: A Randomized Controlled Feasibility Study. *J Ren Nutr*. 2022;32(6):718-25.

20. Chisholm-Burns MA, Spivey CA, Sredzinski E, Butler S. Preliminary results of a behavrioal contract intervention to improve adherence and quality of life among renal transplant recipients. *Am J Transplant*. 2011;2):365.

21. Greenwood SA, Koufaki P, Mercer TH, Rush R, O'Connor E, Tuffnell R, et al. Aerobic or Resistance Training and Pulse Wave Velocity in Kidney Transplant Recipients: A 12-Week Pilot Randomized Controlled Trial (the Exercise in Renal Transplant [ExeRT] Trial). *Am J Kidney Dis*. 2015;66(4):689-98.

22. Hedayati A, Noorbala AA, Khatami SMR. The impact of expressive emotion brief psychotherapy on psychological health of kidney transplant recipients. Iranian Red Crescent *Medical Journal*. 2017;19(3).

23. Henggeler CK, Plank LD, Ryan KJ, Gilchrist EL, Casas JM, Lloyd LE, et al. A Randomized Controlled Trial of an Intensive Nutrition Intervention Versus Standard Nutrition Care to Avoid Excess Weight Gain After Kidney Transplantation: The INTENT Trial. *J Ren Nutr*. 2018;28(5):340-51.

24. Hu S, Xiong R, Hu Q, Li Q. Effects of Nursing Intervention Based on Health Belief Model on Self-Perceived Burden, Drug Compliance, and Quality of Life of Renal Transplant Recipients. *Contrast Media Mol Imaging*. 2022;2022:3001780.

25. Kastelz A, Fernhall B, Wang E, Tzvetanov I, Spaggiari M, Shetty A, et al. Personalized physical rehabilitation program and employment in kidney transplant recipients: a randomized trial. *Transpl Int*. 2021;34(6):1083-92.

26. Lee KL, Peng YL, Chou JL, Lee KT, Chung HM. Economic evaluation of therapeutic drug monitoring services in renal transplant recipients treated with cyclosporine. *Transplant Proc*. 2000;32(7):1801-6.

27. Loftus-Farren KJ, Mahendran AO, Knier T, Mark I, Lord E, Pichardo M, et al. A prospective, randomized, single-blinded trials of mini incision vs standard gibson incision renal transplant recipient operation. *Transplantation*. 2012;10S):832.

28. Mahrova A, Svagrova K, Bunc V, Stollova M, Teplan V. The importance of an early exercise and nutrition intervention among renal transplant recipients. *Kidney Research Clin Pract*. 2012;31(2):A55.

29. Marinho PEM, Rocha LG, Araujo Filho JC, Araujo AXP, Andrade MDA, Taiar R, et al. Effects of whole-body vibration on muscle strength, quadriceps muscle thickness and

functional capacity in kidney transplant recipients: A randomized controlled trial. J *Bodywork Mov Ther*. 2021;26:101-7.

30. Ooms LSS, Minnee RC, Dor F, Kimenai D, Tran KCK, Hartog H, et al. Stenting the ureteroneocystostomy reduces urological complications in kidney transplantation: a noninferiority randomized controlled trial, SPLINT trial. *Transpl Int*. 2020;33(10):1190-8.

31. Riess KJ, Haykowsky M, Lawrance R, Tomczak CR, Welsh R, Lewanczuk R, et al. Exercise training improves aerobic capacity, muscle strength, and quality of life in renal transplant recipients. *Appl Physiol Nutr Metab*. 2014;39(5):566-71.

32. Tzvetanov I, West-Thielke P, D'Amico G, Johnsen M, Ladik A, Hachaj G, et al. A novel and personalized rehabilitation program for obese kidney transplant recipients. *Transplant Proc*. 2014;46(10):3431-7.

33. Yan L, Lin J, Zhao C, Xiong C. The effects of collaborative care model (CCM) guided nursing practice on the self-management and self-efficacy of kidney transplant recipients. *Intern J Clin Experimental* Med. 2021;14(1):356-64.
